# Clues on the origin of post-2000 earthquakes at Campi Flegrei caldera (Italy)

**DOI:** 10.1038/s41598-017-04845-9

**Published:** 2017-06-30

**Authors:** G. Chiodini, J. Selva, E. Del Pezzo, D. Marsan, L. De Siena, L. D’Auria, F. Bianco, S. Caliro, P. De Martino, P. Ricciolino, Z. Petrillo

**Affiliations:** 1 Istituto Nazionale di Geofisica e Vulcanologia, Sezione di Bologna, via D. Creti 12, 40128 Bologna, Italy; 2 Istituto Nazionale di Geofisica e Vulcanologia, Sezione di Napoli Osservatorio Vesuviano, via Diocleziano 328, 80124 Napoli, Italy; 3 Istituto Andalùz de Geofisica, Università de Granada, C/ Profesor Clavera Nº12, Granada, 18071 Spain; 4 ISTerre, CNRS, Université de Savoie Mont Blanc, Campus Scientifique, 73376 Le Bourget du Lac, France; 5 School of Geosciences, Geology and Petroleum Geology, King’s College, University of Aberdeen, Aberdeen, UK; 6 Instituto Volcanológico de Canarias (INVOLCAN), 38400 Puerto de la Cruz, Tenerife, Spain

## Abstract

The inter-arrival times of the post 2000 seismicity at Campi Flegrei caldera are statistically distributed into different populations. The low inter-arrival times population represents swarm events, while the high inter-arrival times population marks background seismicity. Here, we show that the background seismicity is increasing at the same rate of (1) the ground uplift and (2) the concentration of the fumarolic gas specie more sensitive to temperature. The seismic temporal increase is strongly correlated with the results of recent simulations, modelling injection of magmatic fluids in the Campi Flegrei hydrothermal system. These concurrent variations point to a unique process of temperature-pressure increase of the hydrothermal system controlling geophysical and geochemical signals at the caldera. Our results thus show that the occurrence of background seismicity is an excellent parameter to monitor the current unrest of the caldera.

## Introduction

Forecasting the evolution of a volcano in unrest requires interpretation on earthquakes, ground deformation, and volcanic degassing processes^1, 2^. When dealing with restless calderas, this interpretation is challenging. While eruptions do not always follow clear signs of unrest, calderas can erupt with little warning, preceded only by small unrest signals^3, 4^. Due to this complex behaviour and the hazard associated to their large-scale eruptions, calderas are generally considered the most dangerous types of volcanoes.

Starting from 1950’s, Campi Flegrei caldera (CFc) has shown clear signs of reawakening^5^. Since then, a series of inflation episodes of short duration (1–2 years) and abrupt intensity (1.8 m ground uplift in 1983–1984) has interrupted the long deflation phase started after the last eruption (Monte Nuovo eruption, A.D. 1538; ref. 6). This pattern changed at the beginning of the new millennium, when a long, still ongoing period of semi-continuous and accelerating ground uplift has worked in parallel with large variations in the composition of the main fumaroles, and changes in seismicity patterns^7–9^.

**Figure 1:**
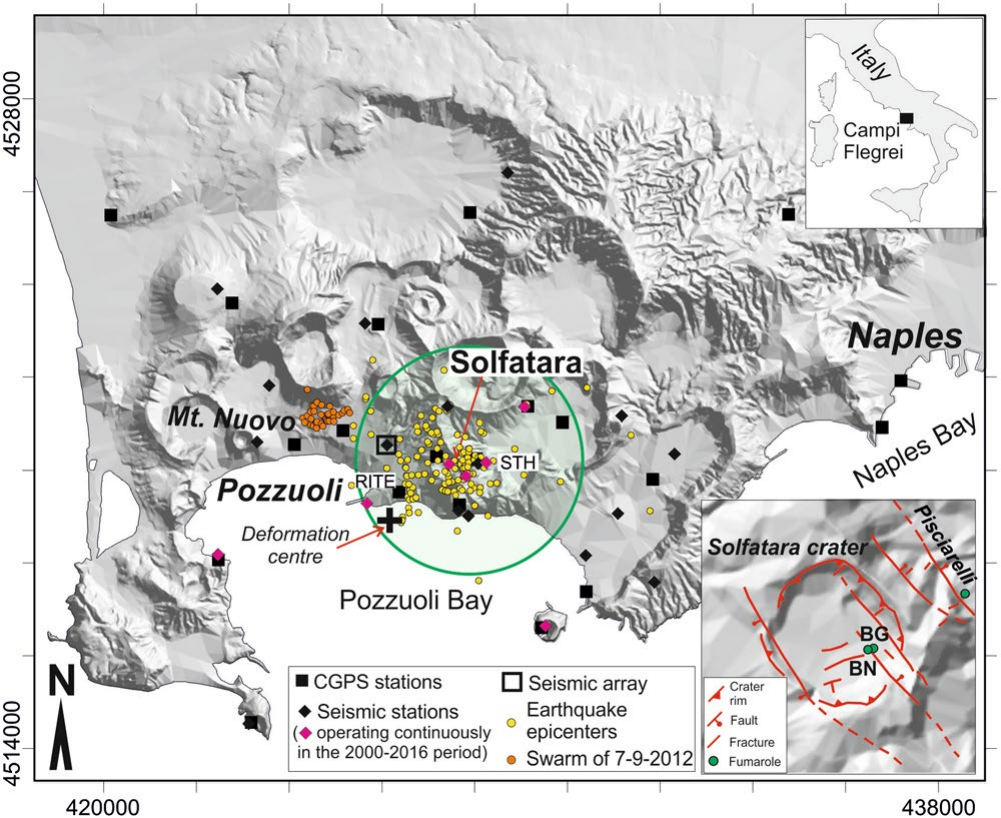
Campi Flegrei caldera and the monitoring system of the Osservatorio Vesuviano-INGV. The map was obtained using the open-access digital elevation model of Italy, TINITALY/01^54^. The seismic and geodetic networks comprise 23 seismic stations, one small aperture seismic array, and 20 continuous GPS stations (CGPS). The map shows the location of the fumaroles that are systematically sampled (BG and BN in Solfatara crater and Pisciarelli, right bottom inset). The green circle is the horizontal section of the computational domain used in the TOUGH2 model. The yellow and orange circles are the post-2000 earthquake epicentres of the best located events^9^. The earthquakes generally occurred in the area of the computational domain of the fluid-dynamic model with the exception of a swarm of events happened on September 2012 (orange circles). Figure generated with Surfer 10 by Golden Software (http://www.goldensoftware.com/products/surfer) and CorelDRAW X5 (http://www.coreldraw.com).

In this work, the CFc seismicity is discussed in combination with the other monitoring parameters, i.e. ground deformation data and the compositions of the main fumaroles located inside Solfatara, the most active zone of the caldera (Fig. 1).

Since 2000 the earthquake occurrence rate and seismic energy release have increased relatively in time, even if both parameters remain low^10–12^ (e.g. max duration magnitude 2.5). Despite the low intensity of earthquakes, the low rate of earthquake occurrence, and the rarity of Long Period (LP) and tremor events many studies have tried to model the mechanisms of the recent CFc seismicity^9, 13–17^. However, the interpretation of the current processes leading to CFc post-2005 activity is mainly based both on ground deformation data^18–20^ and on the evolution of the hydrothermal activity (fluxes and fumarolic compositions^21, 22^). Ground deformation data and measured geochemical parameters show in fact remarkable time-dependent variations^7, 8, 18^.

Our aim is to investigate if and how the source of the current seismicity at CFc is associated with the ground deformation and geochemical signals. Deformations and geochemistry, on the one side, suggest the occurrence of magmatic intrusions^18, 23^ and/or the injection of large amounts of magmatic fluids^7^. On the other side, the ongoing shallow, low-magnitude seismicity of CFc is hardly associated with any magma movement. This is the opinion of the scientists involved in recent elicitation experiments, whose conclusion is that earthquakes at CFc may reveal magma movements only if either deep (>3500 m depth) or energetic (M > 2.5–3) (ref. 24; http://bet.bo.ingv.it/elicitazione/public/). Assessing this type of “correlations - not correlations” among different monitoring parameters has important consequences on the quantification of short-term volcanic hazard^25–27^.

In this work, we first extract earthquake swarms (or seismic clusters) from the seismic catalogue, yielding what we name “background seismicity” of CFc. The background seismicity is then compared with ground deformation and gas geochemical indicators from the monitoring system of the Osservatorio Vesuviano-INGV (Fig. 1, see Methods). Finally, the background seismicity is compared with the results of a recently-published thermo-fluid-dynamic model that simulates the effects of repeated injections of magmatic fluids into the CFc hydrothermal system feeding the fumaroles^7^.

## Results

### Statistics of earthquake sequences: swarm and background events

The 2000–2016 CFc seismicity is mainly characterized by swarm-type occurrence of low-magnitude volcanic quakes (Volcano-tectonic – VT and Long period – LP). The most common events are VT events that show duration-magnitudes lower than 2.5. Almost all VT epicenters are clustered inside a 2500 m radius circle centered at Solfatara crater (Fig. 1) and above the depth of 2000 m. Swarms of LP events occur only occasionally^15, 17^ and are characterized by extremely (and not easily quantifiable) low energy. They are localized (when possible due to the low signal-to-noise ratio) in very small volumes (of the order of 200 m side^15^). On the 30th of January 2015, a short duration (of the order of hours) tremor episode has been detected using small aperture arrays (Fig. 1) and attributed to shallow hydrothermal sources^12, 28^.

In this work, we focus only on VT events because, also due to their small energy, adding LP’s to the used seismic catalogue would have produced some difficulty into the completeness determination. On the other hand, again due to their low energy and to the much lower numbers of LP swarms with respect to the VT events, the bias introduced in neglecting them is inessential for our aims. In particular, we focus on VT events with duration magnitude Md > −0.5 (the catalogue in the years 2000 – to the present is reasonably complete for Md > −1, see supplementary information). Petrosino and coauthors (ref. 29) revised the VT Md scale at CFc using improved path and site transfer functions.The authors calculated moment-magnitudes and Wood-Anderson equivalent magnitudes, finding out a bias (underestimation) of the local scale of the order of a factor 0.6 for the lowest magnitude events. Despite this bias, we use in the present paper the magnitude values routinely calculated and still in use at CFc for sake of continuity with past literature. It is noteworthy that the obtained results do not depend on this choice, because they are dependent on the inter-arrival time of VT events and not on their magnitude.

**Figure 2:**
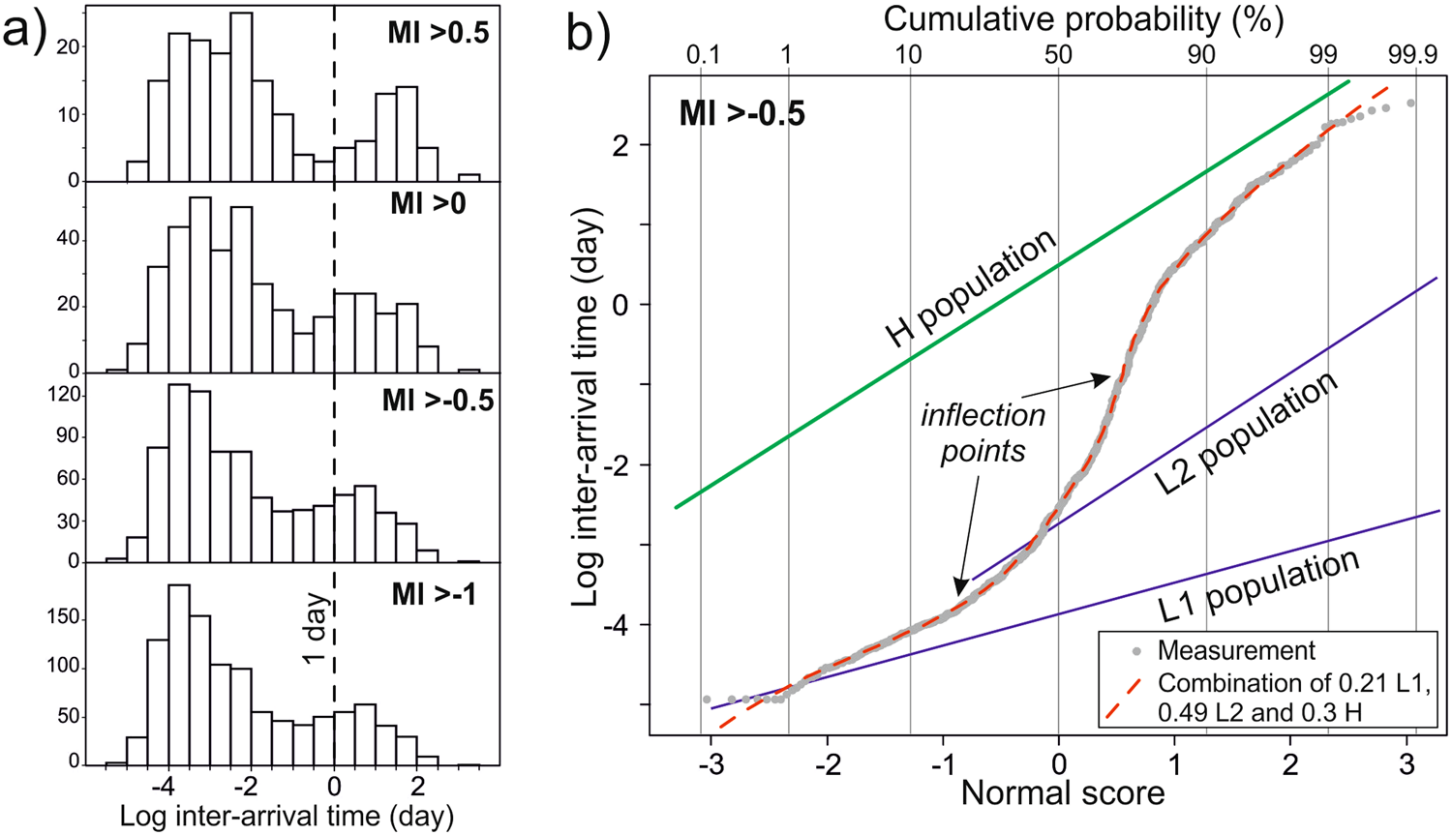
(**a**) Histograms of the log inter-arrival time of Campi Flegrei VT events for different magnitudes. (**b**) Probability plot of log inter-arrival times and partition of the distribution in swarm events (populations L1 and L2) and background events (population H).

While most of the VT events are clustered in space and time (swarms of few hours duration and hundreds of meters lateral extension), a significant number of single events are spatially sparse in time. A bimodal distribution roughly fits the histograms of the earthquake log inter-arrival times at CFc between 2000 and 2016 at all magnitudes (Fig. 2a). The modal values of the two populations are (1) less than 15 minutes for the low inter-arrival time population and (2) more than 3 days for the high inter-arrival time population (Fig. 2a). The two populations correspond to (1) events occurring during volcanic seismic swarms (swarm events) and (2) the sum of seismic swarms plus isolated events that hereafter will be referred as background seismicity. In each of the histograms of Fig. 2a the two populations are roughly divided by a time-interval of approximately 1 day. Practically, the 1-day threshold filters all swarm events out of the CFc earthquake catalogue. The remaining events are what we call background seismicity; their cumulative distribution, which simply corresponds to the sum of the days in which at least one earthquake has occurred, will be referred as CB1.

To separate the swarm events from the background events we applied also a technique originally developed for geochemical data^30^ and more recently used to investigate different populations in soil CO_2_ fluxes^31^. The method is based on plotting the data in a lognormal probability plot (Fig. 2b). A single (*n* = 1) lognormal population would be plotted as a straight line, while *n* overlapping lognormal populations would result in a curve characterized by *n* − *1* inflection points. The observed log inter-arrival times pattern (expressed in log day unit) shows a curve with two inflection points, which describes the theoretical distribution of three (*n* = *3*) overlapping lognormal populations (Fig. 2b). A Monte Carlo approach provides the relevant parameters of the three lognormal populations, i.e. the fraction of each population (*f*), the mean (*μ*), and the standard deviation (*σ*). The results of this test indicate that the observed distribution is given by the overlapping of two low inter-arrival times population L1 and L2 with a high inter-arrival times population H. The estimated parameters *μ*, *σ* and *f* (Table 1) adequately fit the data (Fig. 2b) and were used to compute the probability that each event belongs to the low inter-arrival time populations (either L1 or L2) or to the high inter-arrival times population H by applying the Bayes theorem: Pr(*C*|*x*) = (Pr(*x*|*C*) Pr(*C*))/Pr(*x*). Here *C* is the population of fraction Pr(*C*) = *f* and *x* is each event log inter-arrival time. Pr(*C*|*x*) is computed assuming that *C* follows a log-normal distribution, while Pr(*x*) is computed from the sum of the log-normal data. Finally, Pr(*C*|*x*) is the desired probability that *x* belongs to *C*. In the following, the cumulative of the probabilities that each event belongs to the H population (i.e. the background seismicity) is named CB2.

**Table 1:** Fraction (*f*), mean (*μ*) and standard deviation (*σ*) of the 3 lognormal inter-arrival times populations (Fig. 2b).

Population	*f*	μ	*σ*	Mean (day)
L1	0.21	−3.90	0.39	0.00019
L2	0.49	−2.74	0.94	0.019
H	0.30	0.49	0.92	29

The table reports also the estimated mean (expressed in day) of the correspondent not log distributions.

The third and last approach used to estimate the cumulative distribution of high inter-arrival events is that proposed by ref. 32 for de-clustering seismic catalogues. The method models the rate of earthquakes λ(t) as the sum of the rate v(t) of aftershocks (here swarms) triggered by previous earthquakes (from Omori-Utsu’s and productivity laws^33^) and the rate μ(t) of events triggered by other processes (here background seismicity). The rate μ(t) can be evaluated by subtracting the modelled rate of aftershocks v(t) from the observed rate λ(t). An iterative Expectation-Maximization approach allows to compute, for each earthquake *i*, the background probability as ω_i_ = μ(t_i_)/λ(t_i_). The aftershock rate v(t) is estimated by optimizing a parameterized model made of a combination of both the Omori-Utsu’s and the productivity laws. The approach requires a temporal smoothing of the background probability time series ω_i_ to determine μ(t). We optimize this smoothing parameter by using the Akaike Information Criterion^34^ for several such parameters (for a full description of the method, see ref. 32). The cumulative sum of the background probability ω_i_ will be referred as CB3.

**Figure 3:**
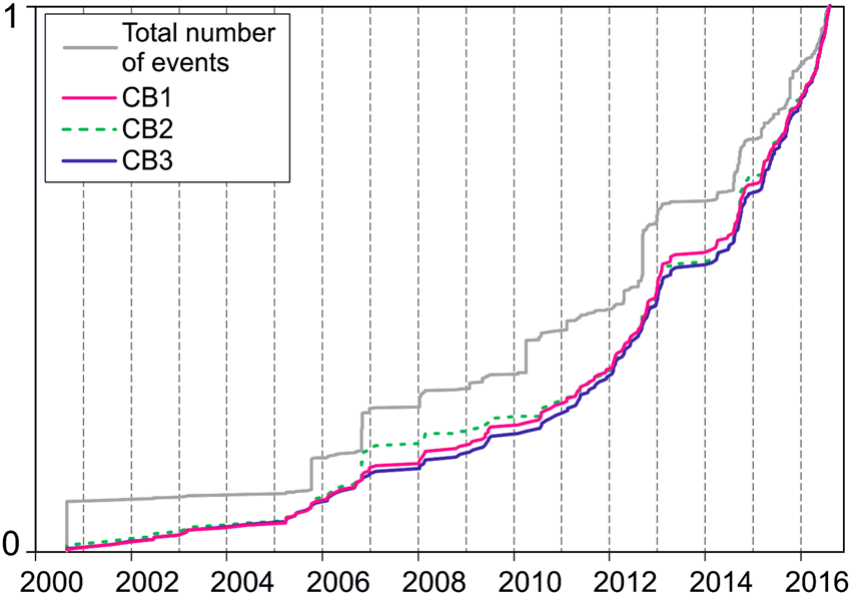
Cumulative curves of total events (magnitude > −0.5) and of de-clustered events (CB1, CB2 and CB3).

CB1, CB2 and CB3 are plotted in Fig. 3 together with the cumulative number of events. The three background curves (CB1, CB2, and CB3) are very similar, and their trend differs from that of the cumulative number of events. It is noteworthy that similar results can be obtained also by standard de-clustering techniques based on the space-time event distribution and not just the time distribution as in the present case^35^.

### Background seismicity, ground deformation and gas geoindicators

**Figure 4:**
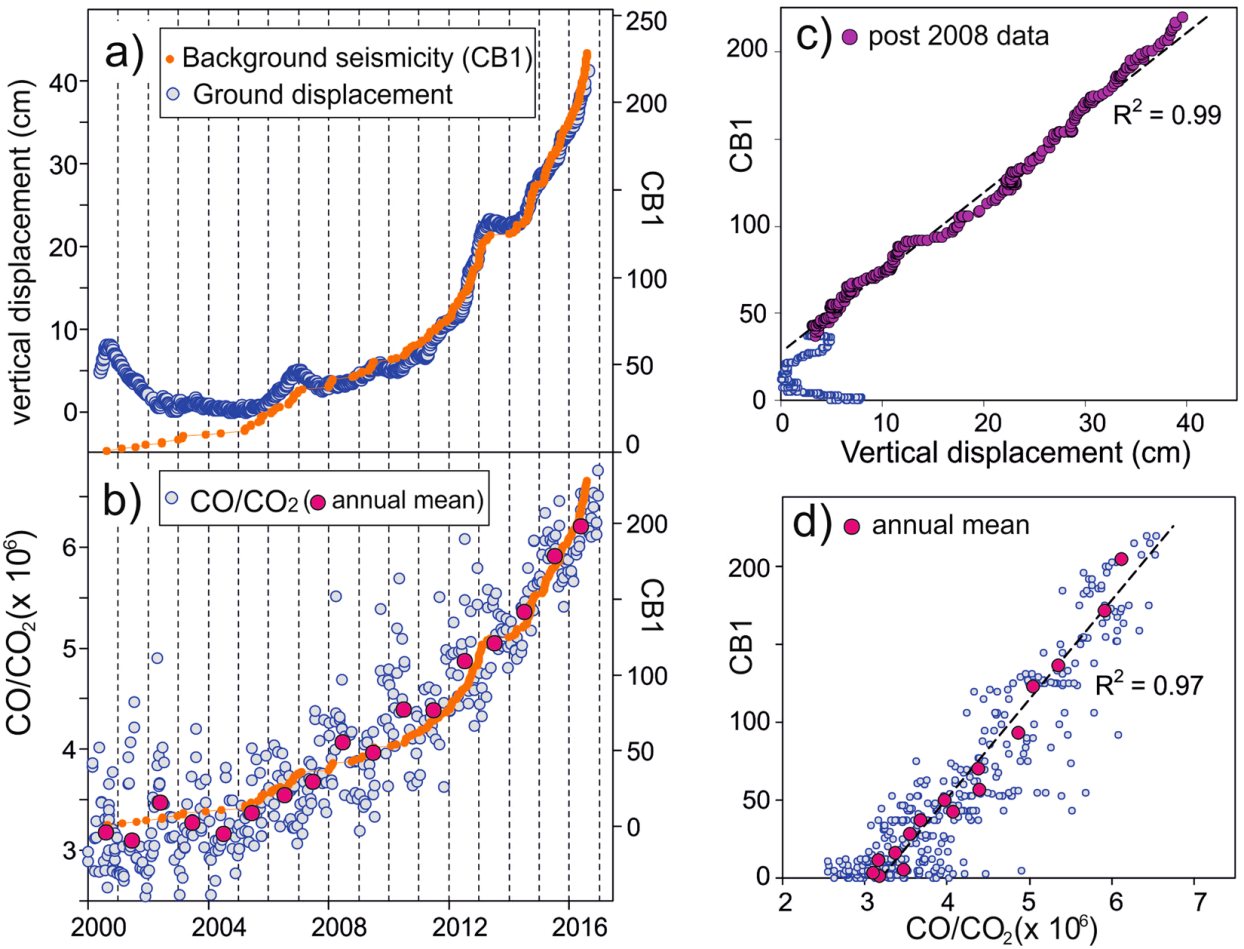
Background seismicity compared with other observations. (**a**) Chronogram of the cumulative background seismicity (orange dots, CB1) and vertical ground displacement at RITE CGPS station; (**b**) chronogram of the cumulative background seismicity (orange dots, CB1) and fumarolic CO/CO_2_ ratios; (**c**) binary plot of the cumulative background seismicity (CB1) vs the vertical ground displacement at RITE CGPS station; (**d**) binary plot of the cumulative background seismicity (CB1) vs the fumarolic CO/CO_2_ ratio (the magenta dots refer to annual mean values of both CO/CO_2_ ratio and CB1).

Here, the background seismicity expressed as the CB1 function is compared with ground deformation and the fumarolic composition time patterns in 2000–2016 (Fig. 4). We used CB1 because this function is the simplest to measure being the cumulative of the events with inter-arrival times higher than 1 day, and because very similar results, practically the same, are obtained substituting CB1 with CB2 or CB3, given the similarity between the curves (Fig. 3).

The chronograms show that CB1 follows the same time pattern of ground deformation (Fig. 4a) and CO/CO_2_ ratio (Fig. 4b). Notably, background seismicity correlates much better to the other observations than other parameters derived from the VT catalogue (e.g. total number of events, swarm events, seismic energy). In particular, since 2008 ground deformation and CB1 are basically identical (Fig. 4c, R^2^ = 0.99). The chronogram (Fig. 4a) indicates that an acceleration has started in 2006, and has been followed by a ~6-years-long period (until 2012–2013) when the signals seem to follow a power-law type curve. The culmination of such period was interpreted as caused by magma intrusion at shallow depth^23^. After one year characterized by no ground deformation and almost null seismicity, both uplift and seismicity drastically increase starting from 2014. Overall, since 2005, the uplift and CB1 signals increase exponentially^7^.

CB1 displays a similar positive correlation with the fumarolic CO/CO_2_ ratio (Fig. 4b,d) that is the most sensitive gas-geothermometer for hydrothermal systems^36, 37^. The points are more scattered in both the chronogram (Fig. 4b) and the binary plot (Fig. 4d) than the vertical displacement. This is likely due to higher analytical uncertainties of the CO/CO_2_ ratio and, possibly, to the occurrence of minor seasonal variations that during the wet seasons cause a cooling of the shallowest parts of the hydrothermal system because of the arrival of cold water. By considering the annual mean (magenta dots) point scattering practically disappears and the seismic and geochemical signal show high correlation in both figures (Fig. 4b,d R^2^ = 0.97). This high correlation between background seismicity and compositional parameter of the fumaroles suggests that the increase of CB1 (and thus the corresponding uplift rates, Fig. 4a) proceeds concurrently with a temperature increase in the subsurface.

### Simulation of the hydrothermal system and background seismicity

**Figure 5:**
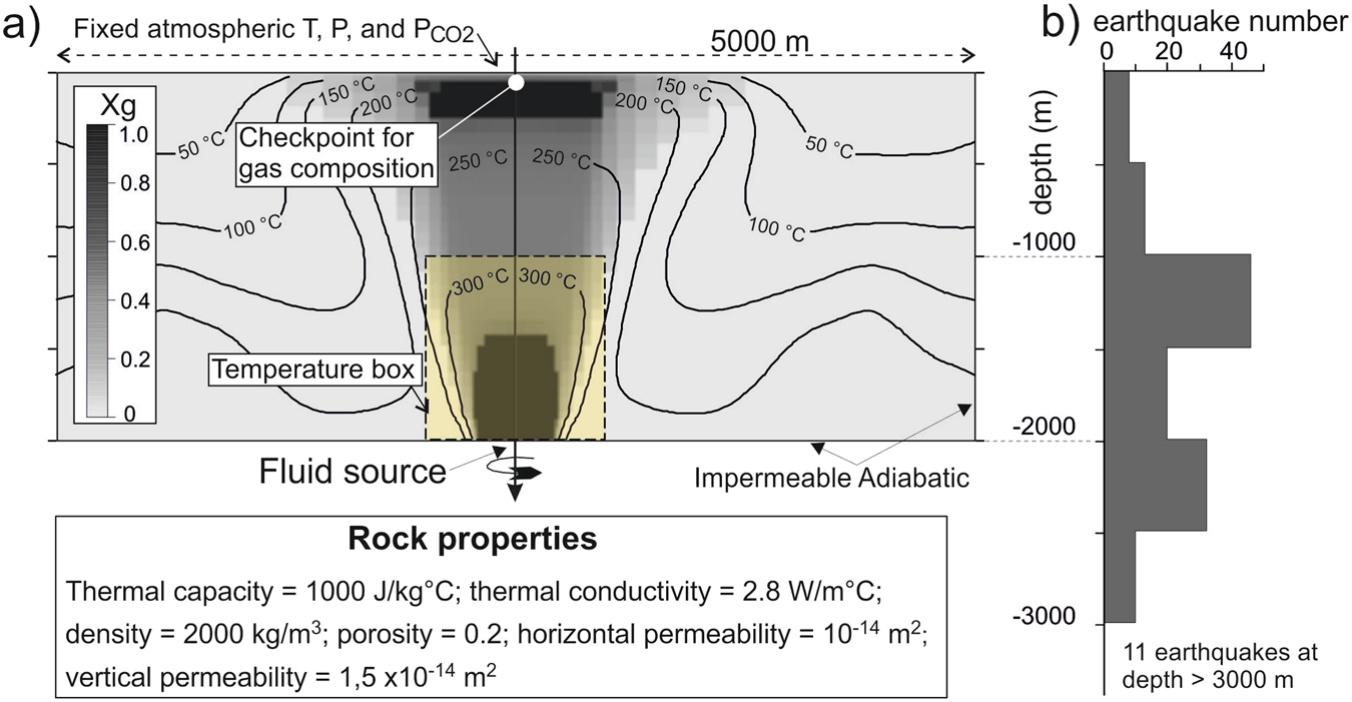
(**a**) The computational domain used in the TOUGH2 simulations. The physical properties of the rocks are homogeneous. The temperature (isolines) and the volumetric gas fraction Xg (different shades of gray) refer to steady-state conditions. The “checkpoint for gas composition” is the zone where the simulated CO_2_/H_2_O is compared with the measured ones^7^. The “Temperature box” (yellow rectangle above the injection zone) is the region where the average temperature is calculated during the simulations (redrawn from ref. 7). (**b**) depth of the best located earthquakes^9^ excluded those occurred on the 7^th^ September 2009 (see Fig. 1). The depth scale in panel (b) corresponds to the one used in panel (a).

At Solfatara, large zones of soil diffuse degassing and fumarolic vents emit an impressive amount of hydrothermal vapour, composed mainly by steam and CO_2_, releasing thermal energy in the order of 100 MW^38^. Recently, a TOUGH2 model^39^ of the hydrothermal processes occurring within the feeding system of Solfatara, possibly controlling the current unrest at CFc, has been proposed^7^. Here we refer to the results of this model whose details are given in the cited reference^7^. Briefly, we used the TOUGH2 geothermal simulator to model the multiphase (gas and liquid) and multi-component (H_2_O and CO_2_) hydrothermal fluid circulation of the system feeding Solfatara fumaroles. The simulations are performed considering a 2D-radial domain (2500 m radius) of 2000 m thick (Fig. 5a), composed of rocks having homogeneous properties. Hydrothermal fluids enter the domain from the bottom (2000 m), in correspondence with the axis of symmetry, and until reaching steady state conditions. The system is then perturbed by injections of high amount of magmatic fluids. The observed CO_2_/CH_4_ and He/CH_4_, which are good indicators of the arrival of a magmatic component at fumaroles^40^, have constrained the timing of 14 episodes of magmatic fluid injections from 1983 to 2014^7^. The magnitude and the CO_2_-H_2_O magmatic composition of each injection were constrained by the measured fumarolic CO_2_/H_2_O and N_2_/He molar ratios, respectively^7^.

**Figure 6:**
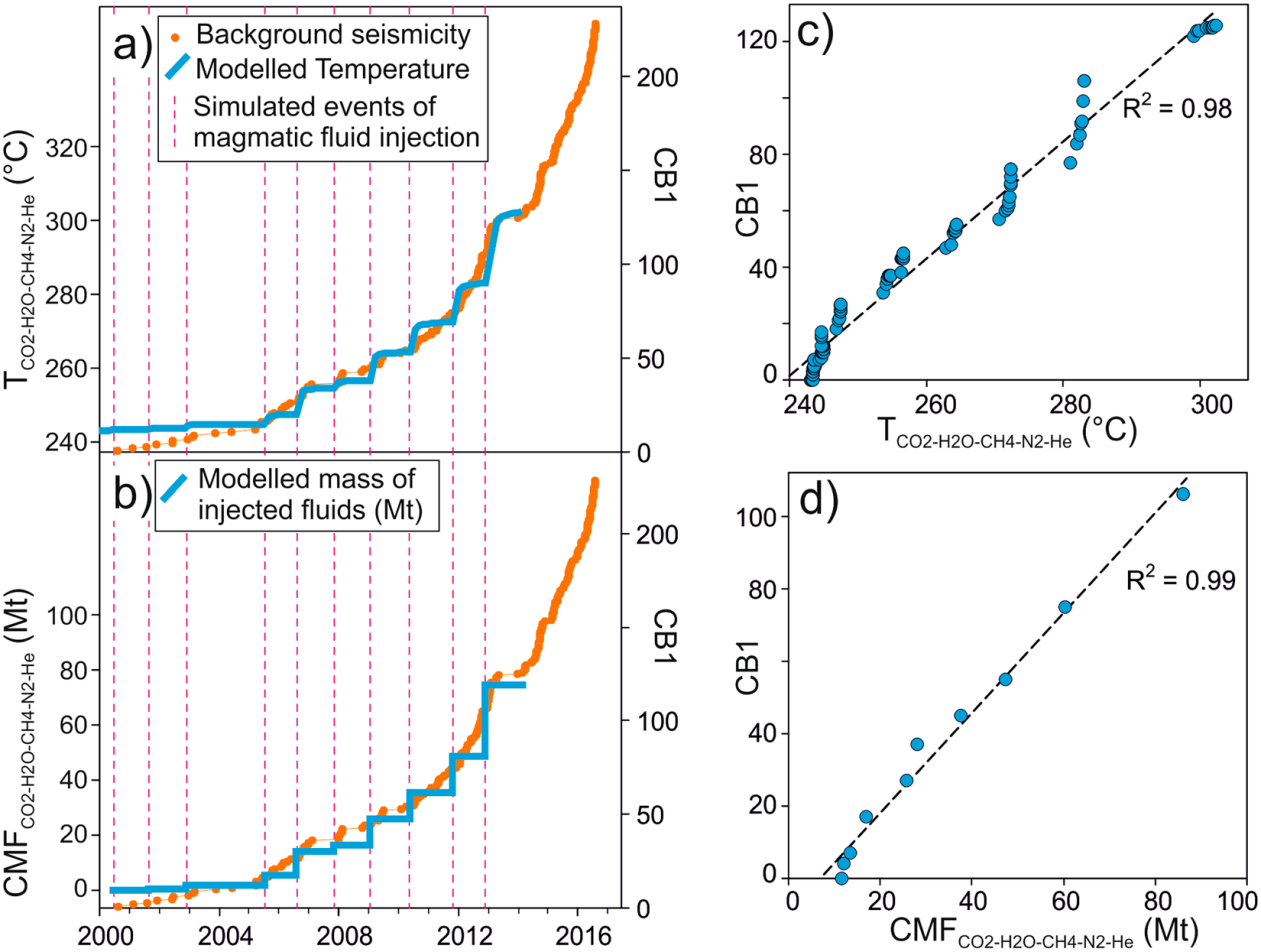
Background seismicity (CB1, see the text) compared with simulation results. (**a**) Chronogram of the cumulative background seismicity (orange dots) and the simulated temperature of the volume of rocks above the magmatic fluid injection zone (T_CO2-H2O-CH4-N2-He_ °C; see Fig. 5a). The vertical magenta dashed lines indicate the time of the simulated episodes of magmatic fluid injection; (**b**) chronogram of the cumulative background seismicity (orange dots) and the cumulative mass of magmatic fluids injected into the hydrothermal system during the simulation (CMF_CO2-H2O-CH4-N2-He_); (**c**) binary plot of CB1 vs T_CO2-H2O-CH4-N2-He_; (**d**) binary plot of CB1 vs CMF_CO2-H2O-CH4-N2-He_.

Here, results of interest are the cumulative mass of magmatic fluids injected into the hydrothermal system (CMF_CO2-H2O-CH4-N2-He_ Mt), and the temperature (T_CO2-H2O-CH4-N2-He_ °C) of the rocks above the injection zone (Fig. 5a) simulated in the 2000–2014 period. The simulated absolute values of CMF_CO2-H2O-CH4-N2-He_ and T_CO2-H2O-CH4-N2-He_ partially depend on the initial steady state conditions (i.e. initial flux and composition of the hydrothermal fluids, rock properties, boundary conditions), however their temporal evolution during the simulation is a complex function of the fumarolic CO_2_-H_2_O-CH_4_-N_2_-He composition only, because these variables constrained the simulation while rock properties and boundary conditions have remained unchanged. Considering that no geophysical data were involved to constrain the simulation, the similarity of the temporal evolutions of CB1, T_CO2-H2O-CH4-N2-He_, and CMF_CO2-H2O-CH4-N2-He_ (Fig. 6a,b), shown here by the high correlation of the seismic and geochemically derived signals (Fig. 6c,d; R^2^ = 0.98 and R^2^ = 0.99, respectively), is thus independent of the input of the model. This correspondence suggests an intimate relation between background seismicity and hydrothermal circulation and supports the reliability of the conceptual model of repeated magmatic fluid injections as the engine of the ongoing crisis of CFc.

## Discussions and Conclusions

Ground deformations, seismicity, and geochemical variations are independent observations, but show the same temporal pattern. They thus point to a unique process controlling the ongoing crisis at CFc. Each observation, and in particular the seismic signal, is in strong correlation with the results (mass of injected fluids and temperature) of a thermo-fluid-dynamic model of repeated injections of high temperature magmatic fluids into the hydrothermal system feeding the fumaroles of Solfatara. These correlations are relevant and robust, as seismic data, geodetic data, and CO/CO_2_ ratios were not used to constrain the numerical model. They indicate that the observed patterns are all likely controlled by the pressure and temperature increase of the hydrothermal system due to repeated, impulsive transfers of high amount of magmatic gases from depth. This control is unsurprising considering that, since 2000, the cumulative seismic energy (~5 × 10^9^ J) is orders of magnitude lower than the thermal energy released by fluid expulsion just at Solfatara (~5 × 10^16^ J assuming a thermal release of 100 MW^38^), and without accounting for the portion of energy lost in heating of the rocks in the subsurface.

In our interpretation, one of the reasons of the coincidence between background seismicity and geochemical simulations is that the computational domain of the model, centered at Solfatara (green circle in Fig 1 and Fig 5a) and 2000 m thick, practically coincides with the volume of rocks affected by the post-2000 seismicity (Figs 1 and 5b). This volume comprises on map a low-attenuation circular area obtained via seismic coda-wave attenuation imaging using the earthquake data accompanying the 1983–84 1.80 m uplift event^41^. The anomaly has a 500 m radius and includes the areas of maximum deformation during the 1983–84 and 2011–2013 unrests. Its centre is located 1000 m SW of the centre of our model and extends at a depth of ~2250 m, thus at the bottom or just below the seismogenic volume (Fig. 5). The anomaly is similar in shape and nature to those associated with ancient magma chambers and/or active intrusions found in other volcanoes. According to general considerations about fluid movements in the magmatic-epithermal environment^42^, the low-attenuation anomaly could correspond to a self-sealed zone of relatively impermeable material. A recent study^43^ discusses the formation of fibrous minerals by intertwining filaments, which may partly concur in the formation of the low attenuation zone evidenced by coda wave tomography. This zone would separate the brittle rocks hosting the hydrothermal circulation from the pressurized plastic region where gases either separated by crystallizing magma^44, 45^ or released by fresh magma accumulate. Episodically, major breaches of the self-sealing zone caused by the increase of magmatic fluid pressure into the plastic zone, would allow the injection of the magmatic gases into the hydrothermal system, exerting a major control on the dynamic of CFc^44–46^. The earthquake-depth histogram shows a maximum earthquake density between 1000 m and 2000 m (Fig. 5b), i.e. at depths compatible with the portion of the computational domain above the zone of magmatic fluids injections (Fig. 1c). Only the 25% of the earthquakes occur instead below the depth of 2000 m, possibly suggesting a progressive transition from brittle to plastic behavior of the rocks associated with very high temperatures^9^. The overlying self-sealed low attenuation zone would separate this deep almost aseismic portion of the caldera from the shallower seismic domain. Here, the temperature and fluid pressure increase caused by magmatic fluid injections would generate sufficient thermo-elastic stress to originate the background VT earthquakes, in accordance with the mechanisms proposed for their origin^47^.

Recently, based solely on mechanical considerations in an elastic-brittle deformation regime, the VT earthquake occurrence at CFc was associated to the brittle partial response of the caldera to the magmatic input^48^. It was proposed that the whole sequence of Campi Flegrei unrests since 1950 belongs to a single, long-term evolutionary trend of accumulating stress and crustal damage, and that the continuation of the trend will favor the progressive approach to eruptive conditions^48^. In this framework, the surprisingly high correlations that we find among independent observations and simulations highlight an additional role of temperature and pressure increase of the hydrothermal system on the process of crustal damage at CFc. In agreement with this interpretation, previous seismological studies suggested the recent occurrence of a transition from elastic to plastic behavior due to fluid saturation and heating of the rocks in the hydrothermal reservoir^9^. Furthermore, a recent analysis of the seismic noise^49^ has discovered a long timescale (2011–2014) decrease of seismic wave velocities in the central part of CFc that is likely related to heating and pressurization. All these evidences point to an increase in the release of H_2_O-rich gases from a depressurizing magmatic system, and the consequent heating of the hydrothermal system^7^. It is worth to note that heating at CFc can be particularly efficient in reducing the rock tensile strength due to the presence of thermally unstable zeolites^50^.

The results of this work have important consequences for the volcanic surveillance of CFc. We show that the occurrence of background seismicity can be considered an excellent parameter to monitor the current unrest of the caldera, since it is highly correlated with ground deformations and geochemical indicators, but simpler to detect. At the same time, any future significant deviation among these parameters may imply significant changes from the current unrest dynamics. These findings must be considered in the framework of recent literature, showing (1) the occurrence of potential recent magmatic intrusions^23^, (2) the increase in magma degassing, pointing to a critical pressure value^7^, and (3) the progressive approach to eruption of the caldera^48^. The need of updating all the short-term forecasting tools presently applied to Campi Flegrei is thus self-evident. This can be done in the framework of new group discussions and consequent elicitations, as those within the updating scheme discussed in ref. 24.

Noteworthy, our new analysis based on the extraction from seismic catalogues of the background seismicity and its comparison with other signals (i.e. ground deformation and gas compositions) can find general applications in understanding the causes of unrest at any volcano, and particularly at calderas.

## Methods

In this section, the data used in the study are briefly illustrated. The data are obtained from the monitoring system of the Osservatorio Vesuviano-INGV (OV). The system consists of several permanent networks, which provide geodetic, seismological and geochemical data, and systematic surveys for gas composition of the fumaroles in the Solfatara crater (Fig. 1, lower right panel).

### Earthquakes

The current permanent seismic network of CFc (Fig. 1, black diamonds) is composed of 18 broadband three-component digital stations, 2 short-period three-components analog stations and three short-period single-component analog ones, for a total of 23 stations. Data transmission in real time to the OV Monitoring Center is realized by different systems such as UHF, Wi-Fi radio links, TCP/IP client-server applications. The CFc earthquake catalog used in this work (supplementary dataset 1) contains a data set of about 1800 VT earthquakes recorded between 2000 and July 2016, with magnitude ranging between −2.5 and 2.5. In 2000, the permanent seismic network of CFc was composed of 8 short-period analog stations and 1 broadband digital one, for a total of 9 stations. Seven of these stations have operated continuously until today and represent the initial core of the present network (Fig. 1). In particular, the STH station is adopted as reference station for CFc seismicity because of its closeness to the Solfatara area where the post-2000 seismicity concentrates. Starting from 2005, more stations were added to the CFc seismic network increasing the number of broadband digital stations and covering a more wide area, reaching the present configuration. The network development has improved the hypocenter locations quality but did not add significant effects on the detection capability because the stations distribution provided, already in the early 2000, an appropriate coverage of the area interested by 2000–2016 seismicity.

### Ground deformation

Ground deformations are monitored through the NeVoCGPS (Neapolitan Volcanoes Continuous GPS) network. The network provides measurements of the 3D time changes in the position of 36 permanent stations, located in the Neapolitan volcanic district and surrounding area^51, 52^. At present, 20 of these continuous GPS (CGPS) stations are operating at CFc (Fig. 1). A full description of CGPS network and of processing strategies, as well as the 2000–2013 complete database are reported in a previous work^51^. The supplementary dataset 2 reports the updated data to July 2016 of the vertical displacement at RITE GPS station. The RITE GPS station (Fig. 1) is commonly adopted as reference station for CFc because it is closest to the zone of maximum vertical displacement. Here, we assume this station as representative of the time pattern of ground deformations at CFc. We note, however, that the temporal pattern of the vertical deformation is very similar at all the GPS stations^51^.

### Chemical composition of fumaroles

In the last ten years, time series of chemical compositions of Solfatara fumaroles (BG, BN and Pisciarelli, Fig. 1) were published in different works (e.g. ref. 7). Analytical methodologies and uncertainties are described in ref. 53. Here, we consider the time series of the CO/CO_2_ ratio measured at BG and BN fumaroles updated to July 2016. This ratio is an excellent indicator of the temperature variations at depth^36^.

### Data availability

All relevant data are available from the authors.

## Electronic supplementary material

Data sets
