# Supplementary material for: Clues on the origin of post-2000 earthquakes at Campi Flegrei caldera (Italy)
Source: Sci Rep. 2017 Jun 30;7:4472. doi: 10.1038/s41598-017-04845-9 (PMC5493613; doi:10.1038/s41598-017-04845-9)
Supplement: Supplementary file 1 — Data sets [file 41598_2017_4845_MOESM1_ESM.pdf]

Clues on the origin of post-2000 earthquakes at Campi Flegrei caldera (Italy)

Chiodini G., Selva J., Del Pezzo E., Marsan D., De Siena L., D'Auria L., Bianco F., Caliro S., De Martino P., Ricciolino P., Petrillo Z.

Supplementary information

	page
1) Supplementary dataset1: Campi Flegrei earthquakes 2000_2016	2
2) Supplementary dataset2: Vertical displacement at RITE CGPS 2000_2016	30

Supplementary dataset 1: Campi Flegrei earthquakes 2000_2016

Completeness of the catalogue

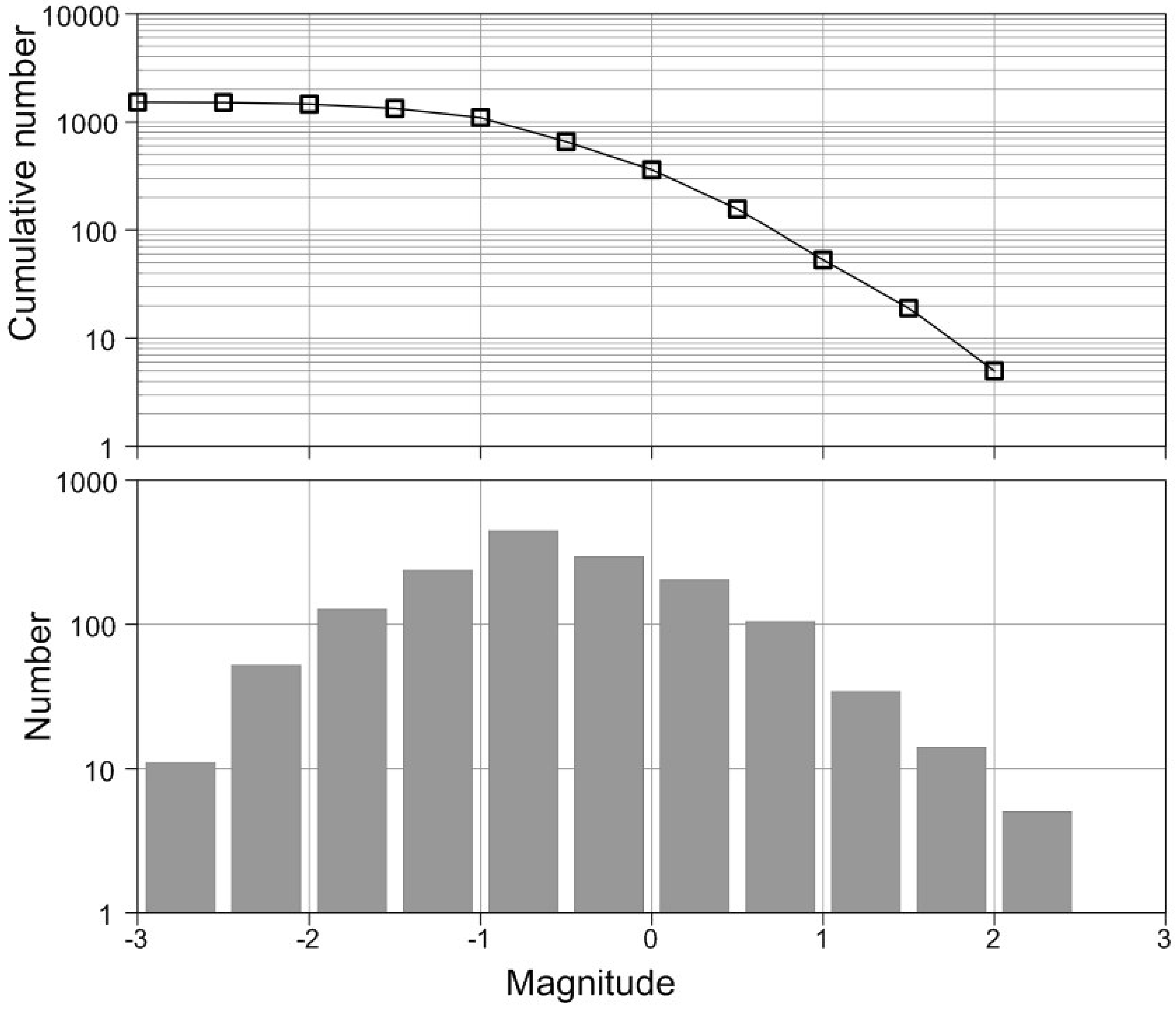

The frequency size distribution is reported in terms of cumulative number of events with M > x (upper panel), and number of events in each magnitude interval (lower panel); the catalogue is reasonably complete for magnitudes > -1.

Time is expressed in Coordinated Universal Time. Duration Magnitude (Md) = -2.46 +2.82 log t where t is the duration measured from the P-wave onset to the time point in which signal amplitude equals the noise-amplitude; N.D. Not determined

Date (yyyy-mm-dd), Time (hh:mm:ss.s), Md

22/08/2000	13:38:39.7	0.6
22/08/2000	13:46:21.7	0.9
22/08/2000	13:58:21.7	-0.5
22/08/2000	14:06:41.1	-0.1
22/08/2000	14:07:39.5	-0.1
22/08/2000	14:14:10.6	0
22/08/2000	14:17:01.4	1.4
22/08/2000	14:25:04.4	0.9
22/08/2000	14:25:52.3	-0.3
22/08/2000	14:25:58.2	-0.5
22/08/2000	14:30:11.3	0.7
22/08/2000	14:30:53.0	-0.3
22/08/2000	14:36:22.5	-0.1
22/08/2000	14:39:21.4	-0.5
22/08/2000	14:42:11.6	0
22/08/2000	14:43:21.5	-0.5
22/08/2000	14:44:53.3	0.1
22/08/2000	14:44:54.0	-0.3
22/08/2000	14:56:37.0	-0.1
22/08/2000	14:58:44.0	-0.5
22/08/2000	15:01:15.7	-0.5
22/08/2000	15:01:23.5	-0.5
22/08/2000	15:02:38.1	-0.5
22/08/2000	15:05:56.8	1.6
22/08/2000	15:06:40.6	-0.3
22/08/2000	15:17:53.6	0.7
22/08/2000	15:28:40.6	-0.3
22/08/2000	15:29:27.6	0.4
22/08/2000	15:36:39.0	0.2
22/08/2000	15:37:00.6	-0.1
22/08/2000	15:39:42.8	-1.1
22/08/2000	15:44:24.4	0.9
22/08/2000	15:45:11.1	-0.8
22/08/2000	15:46:37.0	-0.8
22/08/2000	15:49:44.2	-1.1
22/08/2000	15:53:50.3	0.5
22/08/2000	15:58:02.3	2.2
22/08/2000	16:01:39.2	0
22/08/2000	16:08:51.3	-0.8
22/08/2000	16:15:46.0	-1.1
22/08/2000	16:15:51.6	0
22/08/2000	16:43:04.8	0.7
22/08/2000	16:50:22.0	0
22/08/2000	16:51:23.2	0
22/08/2000	16:55:41.2	-0.3
22/08/2000	17:05:35.2	0
22/08/2000	17:06:02.7	1.7
22/08/2000	17:08:57.6	0
22/08/2000	17:09:08.0	0
22/08/2000	17:20:25.5	0
22/08/2000	17:22:26.3	0.7
22/08/2000	17:31:35.5	1.9
22/08/2000	17:41:27.7	0
22/08/2000	17:42:43.2	0
22/08/2000	17:44:54.1	-0.5
22/08/2000	17:47:22.3	-0.5
22/08/2000	17:57:19.9	0.1
22/08/2000	18:00:43.2	-0.8
22/08/2000	18:01:20.2	0
22/08/2000	18:09:04.8	0.1
22/08/2000	18:11:00.8	0
22/08/2000	18:20:54.5	1.4

22/08/2000	18:25:02.4	-0.3
22/08/2000	18:33:02.3	0
22/08/2000	18:51:45.6	0
22/08/2000	18:54:22.1	-1.1
22/08/2000	19:00:26.2	-0.8
22/08/2000	19:03:31.8	-0.3
22/08/2000	19:06:26.1	-1.6
22/08/2000	19:06:30.4	-0.3
22/08/2000	19:23:11.8	0.4
22/08/2000	19:24:18.4	0
22/08/2000	19:42:56.7	0
22/08/2000	19:43:20.9	-0.5
23/08/2000	10:40:37.0	-0.8
23/08/2000	11:26:31.0	1.2
23/08/2000	11:59:34.7	-0.5
23/08/2000	12:27:38.9	1.3
23/08/2000	13:59:21.0	1.1
23/08/2000	13:59:51.0	0.4
23/08/2000	15:41:43.9	-0.5
23/08/2000	15:58:13.5	0.4
23/08/2000	16:46:44.2	0.9
23/08/2000	17:12:57.0	1.7
23/08/2000	17:42:22.3	0
23/08/2000	17:42:29.8	0
23/08/2000	18:30:41.8	1
23/08/2000	19:04:02.7	1.1
23/08/2000	19:57:31.4	0
23/08/2000	20:11:05.3	0.4
20/02/2001	06:20:47.5	0.9
07/08/2001	01:34:50.7	0.8
06/12/2001	14:08:11.1	-0.1
13/06/2002	12:15:44.0	0.1
13/06/2002	13:45:41.6	-0.1
19/06/2002	12:00:34.5	0.2
30/10/2002	10:25:06.9	N.D.
25/12/2002	06:35:59.2	1
31/01/2003	03:23:36.9	N.D.
18/02/2003	15:32:23.2	1.7
02/03/2003	06:58:11.3	0.9
26/11/2003	17:13:26.3	1.4
07/06/2004	04:40:29.7	0.2
22/03/2005	17:08:20.1	0.4
24/03/2005	08:53:34.0	N.D.
24/03/2005	16:04:09.2	-0.5
27/03/2005	00:56:02.9	-0.5
28/05/2005	21:26:45.3	0.8
08/06/2005	01:08:12.3	0.4
25/08/2005	09:37:59.3	1.1
24/09/2005	08:57:27.3	0.6
05/10/2005	10:07:19.2	-0.5
05/10/2005	10:14:08.8	-0.8
05/10/2005	10:14:16.8	-0.1
05/10/2005	10:39:41.4	-0.5
05/10/2005	11:13:15.1	N.D.
05/10/2005	11:13:45.0	-0.3
05/10/2005	11:28:37.9	-0.1
05/10/2005	11:36:17.1	N.D.
05/10/2005	11:36:18.8	N.D.
05/10/2005	11:53:13.2	0.5
05/10/2005	12:07:46.0	-0.8
05/10/2005	12:13:17.5	-0.5
05/10/2005	12:14:10.6	-0.5
05/10/2005	12:16:24.0	N.D.
05/10/2005	12:16:51.2	N.D.
05/10/2005	12:38:34.8	0.7
05/10/2005	12:39:58.6	N.D.
05/10/2005	12:40:07.6	-0.3
05/10/2005	13:20:11.3	-0.3
05/10/2005	13:20:58.2	N.D.
05/10/2005	13:21:00.3	N.D.

05/10/2005	13:21:07.1	0.2
05/10/2005	13:33:25.2	0.6
05/10/2005	13:33:36.1	-1.1
05/10/2005	13:33:51.0	N.D.
05/10/2005	13:43:27.8	N.D.
05/10/2005	13:55:54.3	N.D.
05/10/2005	14:03:57.9	-0.5
05/10/2005	14:09:49.5	0.7
05/10/2005	14:34:41.9	-0.1
05/10/2005	14:39:19.5	0.1
05/10/2005	14:39:31.5	N.D.
05/10/2005	14:51:08.6	0.6
05/10/2005	15:22:04.7	0.2
05/10/2005	15:22:23.2	0.6
05/10/2005	15:26:40.8	-0.1
05/10/2005	15:39:26.7	0.2
05/10/2005	15:41:22.4	-0.5
05/10/2005	15:41:40.0	-0.5
05/10/2005	15:44:53.5	-0.5
05/10/2005	15:49:28.0	0.5
05/10/2005	16:00:48.6	0.7
05/10/2005	16:18:01.9	1.1
05/10/2005	16:20:14.3	N.D.
05/10/2005	16:21:27.1	N.D.
05/10/2005	16:21:30.5	0.6
05/10/2005	16:29:59.0	0.8
05/10/2005	16:30:00.5	N.D.
05/10/2005	16:32:50.3	-0.1
05/10/2005	16:42:55.6	0
05/10/2005	16:42:59.5	-0.5
05/10/2005	16:43:18.3	N.D.
05/10/2005	16:45:07.7	0.6
05/10/2005	16:45:29.9	-1.1
05/10/2005	16:45:42.8	-1.1
05/10/2005	16:53:09.5	-0.3
05/10/2005	16:53:18.1	-0.3
05/10/2005	16:54:02.2	0.1
05/10/2005	16:56:29.0	-0.1
05/10/2005	16:56:51.3	N.D.
05/10/2005	16:57:06.3	-0.5
05/10/2005	17:00:28.9	-0.3
05/10/2005	17:00:48.3	N.D.
05/10/2005	17:01:00.0	-0.1
05/10/2005	17:01:49.5	N.D.
05/10/2005	17:06:32.7	N.D.
05/10/2005	17:09:44.7	0.2
05/10/2005	17:10:39.5	N.D.
05/10/2005	17:10:44.5	N.D.
05/10/2005	17:10:50.4	N.D.
05/10/2005	17:11:07.2	N.D.
05/10/2005	17:11:09.0	N.D.
05/10/2005	17:11:16.1	N.D.
05/10/2005	17:11:21.1	0.2
05/10/2005	17:11:33.8	N.D.
05/10/2005	17:16:10.7	-0.5
05/10/2005	17:16:29.4	N.D.
05/10/2005	17:26:23.9	-0.8
05/10/2005	17:27:31.0	-0.5
05/10/2005	17:31:22.9	0.6
05/10/2005	17:32:14.8	-0.8
05/10/2005	17:46:53.4	N.D.
05/10/2005	17:46:56.9	-0.1
05/10/2005	17:50:01.9	N.D.
07/10/2005	20:39:36.4	0.1
08/10/2005	13:41:05.6	0.2
05/11/2005	06:16:18.4	1.2
29/01/2006	19:52:03.0	0.6
06/02/2006	08:33:17.1	-0.5
06/02/2006	09:33:14.6	0.4
01/03/2006	14:57:00.9	0.7

08/04/2006	07:09:32.9	0.9
08/04/2006	11:00:09.8	-0.5
08/04/2006	11:00:17.6	-1.6
23/05/2006	02:46:58.5	0.2
29/08/2006	00:47:11.2	0.6
05/09/2006	20:20:53.7	0.4
13/09/2006	01:12:04.7	0.5
13/09/2006	01:12:25.0	-0.5
13/09/2006	01:12:31.3	-1.1
19/10/2006	22:11:27.9	-1.1
19/10/2006	22:59:44.6	-0.5
19/10/2006	23:10:31.1	0.2
19/10/2006	23:15:59.8	-0.8
20/10/2006	01:36:02.4	-0.5
20/10/2006	09:51:11.8	0.4
20/10/2006	09:51:12.3	-1.1
20/10/2006	16:50:50.8	-0.1
20/10/2006	21:56:47.8	-0.3
20/10/2006	22:27:40.8	-0.3
20/10/2006	22:29:13.2	-0.3
20/10/2006	23:22:07.8	-0.1
20/10/2006	23:55:16.5	-0.5
21/10/2006	01:11:58.1	-0.5
21/10/2006	10:28:18.2	-0.8
21/10/2006	11:54:58.8	-1.1
21/10/2006	14:12:01.0	-0.3
21/10/2006	18:01:45.2	-0.3
21/10/2006	23:44:07.8	-0.1
22/10/2006	02:06:23.0	-0.5
22/10/2006	02:06:35.9	-0.5
22/10/2006	02:20:22.9	-0.3
22/10/2006	03:06:39.5	-1.1
22/10/2006	05:55:20.1	-0.5
22/10/2006	06:05:05.0	0.5
22/10/2006	06:20:21.2	-0.8
22/10/2006	22:17:51.9	0
22/10/2006	22:17:56.2	N.D.
22/10/2006	22:17:57.6	N.D.
22/10/2006	22:17:59.6	N.D.
22/10/2006	22:18:09.1	N.D.
22/10/2006	22:18:11.9	N.D.
22/10/2006	22:18:13.5	-0.1
22/10/2006	23:21:16.3	-1.1
22/10/2006	23:21:22.5	-0.5
23/10/2006	08:04:27.5	-1.1
23/10/2006	08:06:07.3	-1.1
23/10/2006	08:06:30.5	N.D.
23/10/2006	08:06:33.1	0.1
23/10/2006	08:06:49.6	0.1
23/10/2006	08:06:59.7	-0.5
23/10/2006	08:07:33.6	0.8
23/10/2006	08:08:17.1	0.6
23/10/2006	08:09:45.2	0.7
23/10/2006	08:10:36.3	0.6
23/10/2006	08:11:50.4	0.7
23/10/2006	08:13:35.6	0.4
23/10/2006	08:18:16.3	0.6
23/10/2006	17:40:47.8	-0.3
23/10/2006	17:46:35.7	0.1
23/10/2006	17:54:09.4	0.4
25/10/2006	15:08:27.8	-2
25/10/2006	15:08:30.0	-2
25/10/2006	15:16:34.2	-2
25/10/2006	15:16:36.7	-2
25/10/2006	15:16:41.7	-2
25/10/2006	15:24:26.7	N.D.
25/10/2006	15:37:13.3	-0.5
25/10/2006	16:00:30.1	-2
25/10/2006	16:00:32.3	-2
25/10/2006	16:00:57.3	-2

25/10/2006	16:00:59.4	-2
25/10/2006	16:01:13.9	-2
25/10/2006	16:01:14.9	-2
25/10/2006	16:01:17.7	-2
25/10/2006	16:01:48.1	-2
25/10/2006	17:57:52.3	-2
25/10/2006	17:57:54.8	-2
25/10/2006	19:30:49.0	-2
25/10/2006	20:41:22.9	-2
25/10/2006	21:19:19.0	-2
25/10/2006	21:20:19.1	-2
25/10/2006	21:22:58.8	-2
25/10/2006	21:23:10.3	-2
25/10/2006	22:18:40.9	0.2
26/10/2006	01:25:20.2	-0.3
26/10/2006	01:38:55.1	-0.1
26/10/2006	03:19:38.7	-0.3
26/10/2006	03:42:56.5	0
26/10/2006	03:53:46.9	-2
26/10/2006	03:53:49.2	-2
26/10/2006	03:56:26.7	-0.5
26/10/2006	03:56:36.0	-1.1
26/10/2006	04:00:42.6	-2
26/10/2006	04:01:00.7	-2
26/10/2006	04:01:20.1	-2
26/10/2006	04:01:35.1	-0.8
26/10/2006	04:01:35.2	-2
26/10/2006	04:02:44.2	-0.3
26/10/2006	04:03:47.2	-0.5
26/10/2006	04:04:25.0	-0.5
26/10/2006	04:04:41.7	-0.8
26/10/2006	04:05:04.7	-0.8
26/10/2006	04:05:12.2	-2
26/10/2006	04:05:26.9	-2
26/10/2006	04:05:37.0	-0.8
26/10/2006	04:05:52.2	-2
26/10/2006	04:08:28.7	-0.8
26/10/2006	04:09:09.2	-2
26/10/2006	04:09:46.8	-0.8
26/10/2006	04:09:51.7	-2
26/10/2006	04:09:57.8	-2
26/10/2006	04:10:00.3	-0.5
26/10/2006	04:10:13.8	0.1
26/10/2006	04:10:55.6	-2
26/10/2006	04:11:14.3	-2
26/10/2006	04:11:25.5	-0.5
26/10/2006	04:11:46.3	-0.5
26/10/2006	04:12:19.6	-0.5
26/10/2006	04:13:36.2	-0.5
26/10/2006	04:16:10.0	-0.8
26/10/2006	04:16:18.4	0.1
26/10/2006	04:16:31.4	-2
26/10/2006	04:17:02.0	-2
26/10/2006	04:18:22.0	-2
26/10/2006	04:20:52.1	-2
26/10/2006	04:20:58.6	-2
26/10/2006	04:21:18.9	-2
26/10/2006	04:21:20.2	-2
26/10/2006	04:21:23.3	-2
26/10/2006	04:42:53.5	-2
26/10/2006	04:42:56.7	-2
26/10/2006	04:43:04.9	-0.8
26/10/2006	04:46:56.0	-2
26/10/2006	04:47:08.0	-0.1
26/10/2006	04:49:41.4	-1.1
26/10/2006	04:53:10.3	0.5
26/10/2006	21:30:47.3	-0.8
26/10/2006	22:48:44.6	-0.8
26/10/2006	22:49:38.8	-0.1
27/10/2006	02:09:55.1	-0.5

27/10/2006	05:53:20.7	-2
27/10/2006	07:46:15.3	-0.5
27/10/2006	08:15:06.5	-2
27/10/2006	08:15:11.5	-1.1
27/10/2006	08:25:11.9	-2
27/10/2006	09:04:31.6	N.D.
27/10/2006	09:04:34.2	-1.1
27/10/2006	09:07:45.3	-1.1
27/10/2006	09:09:09.1	-2
27/10/2006	09:10:32.3	-0.8
27/10/2006	09:59:19.9	-1.1
27/10/2006	09:59:23.3	-1.1
27/10/2006	10:00:00.3	-1.1
27/10/2006	10:51:20.9	-2
27/10/2006	10:51:22.6	-0.8
27/10/2006	11:48:33.8	-1.1
27/10/2006	12:33:32.6	-1.1
27/10/2006	12:53:12.1	N.D.
27/10/2006	14:08:53.0	-2
27/10/2006	16:16:47.6	-0.3
28/10/2006	17:58:03.9	0.2
09/11/2006	00:33:42.1	-0.3
20/12/2006	23:53:05.7	1.1
21/12/2006	22:19:31.6	N.D.
21/12/2006	22:24:33.7	-0.1
21/12/2006	22:25:16.9	0
21/12/2006	22:25:31.8	1.4
21/12/2006	22:26:46.4	-0.1
21/12/2006	22:42:16.0	1.1
24/12/2006	03:19:02.8	-0.3
08/02/2007	01:07:41.4	0.5
03/01/2008	01:03:38.8	-0.3
05/01/2008	19:06:13.7	0.1
05/01/2008	19:07:29.2	-0.8
05/01/2008	19:07:38.4	-0.3
05/01/2008	19:08:09.2	-0.8
05/01/2008	19:08:28.9	N.D.
05/01/2008	19:08:31.3	-0.8
05/01/2008	19:08:40.9	-0.8
05/01/2008	19:09:26.3	-0.3
05/01/2008	19:09:37.4	-1.1
05/01/2008	19:09:55.1	-1.1
05/01/2008	19:09:37.4	-0.3
05/01/2008	19:09:58.7	-0.3
05/01/2008	19:10:07.6	-0.3
05/01/2008	19:10:20.6	-1.6
05/01/2008	19:10:26.7	-1.6
05/01/2008	19:10:43.7	-1.1
05/01/2008	19:11:04.0	N.D.
05/01/2008	19:11:05.9	-0.3
05/01/2008	19:11:27.2	-0.8
05/01/2008	19:11:39.1	N.D.
05/01/2008	19:11:41.3	-0.8
05/01/2008	19:11:50.5	-0.3
05/01/2008	19:13:00.5	-0.3
05/01/2008	19:13:15.1	-0.8
05/01/2008	19:13:42.5	N.D.
05/01/2008	19:13:45.0	-0.5
05/01/2008	19:13:51.4	-1.1
05/01/2008	19:13:57.6	-0.8
05/01/2008	19:14:03.5	-0.5
05/01/2008	19:14:16.7	-1.1
05/01/2008	19:14:23.2	-0.5
05/01/2008	19:14:42.9	-0.8
05/01/2008	19:15:52.4	-1.1
05/01/2008	19:16:22.5	-1.1
05/01/2008	19:17:28.0	N.D.
05/01/2008	19:17:56.3	0.7
05/01/2008	19:19:44.4	-0.8
05/01/2008	19:19:52.8	-0.8

05/01/2008	19:20:40.2	-0.3
05/01/2008	19:22:21.5	N.D.
05/01/2008	19:22:24.0	N.D.
05/01/2008	19:22:24.9	-0.3
05/01/2008	19:23:27.4	-0.8
05/01/2008	19:24:12.9	-0.8
05/01/2008	19:25:31.3	-0.3
20/01/2008	00:54:58.9	N.D.
20/01/2008	00:55:01.2	0.1
20/01/2008	00:55:17.6	0.4
20/01/2008	00:55:29.5	0.1
20/01/2008	00:57:13.1	-0.5
20/01/2008	01:00:32.0	0.1
04/02/2008	20:44:33.7	-0.1
04/02/2008	20:46:29.4	N.D.
04/02/2008	20:46:34.6	1.2
13/02/2008	11:50:30.0	-0.3
19/02/2008	08:40:48.9	0.4
19/02/2008	12:48:41.3	1.6
10/10/2008	11:56:03.7	N.D.
10/10/2008	11:56:59.4	0.5
10/10/2008	11:57:43.3	0.1
10/11/2008	03:01:17.2	-0.1
23/01/2009	05:15:36.9	-1.1
23/01/2009	05:16:03.4	-1.1
23/01/2009	05:16:31.9	-0.8
23/01/2009	05:16:43.8	-0.5
23/01/2009	05:16:56.3	-1.1
23/01/2009	05:17:00.3	-1.1
23/01/2009	05:17:15.5	-1.6
23/01/2009	05:17:24.7	N.B.
23/01/2009	05:17:27.0	N.B.
23/01/2009	05:17:29.4	-1.6
23/01/2009	05:17:33.7	-1.6
23/01/2009	05:17:39.4	-1.1
23/01/2009	05:17:44.3	-1.6
23/01/2009	05:17:50.6	-1.6
23/01/2009	05:18:08.0	-1.1
23/01/2009	05:18:14.8	-0.5
23/01/2009	05:18:25.9	-1.6
23/01/2009	05:18:35.7	-1.6
23/01/2009	05:18:44.4	-1.1
23/01/2009	05:18:51.3	N.B.
23/01/2009	05:21:08.9	-1.1
23/01/2009	05:21:17.2	-1.6
23/01/2009	05:21:24.8	N.B.
23/01/2009	05:21:31.7	N.B.
23/01/2009	05:21:38.6	N.B.
23/01/2009	05:21:43.3	N.B.
23/01/2009	05:21:49.7	-1.1
23/01/2009	05:22:02.1	-1.1
23/01/2009	05:22:08.1	N.B.
23/01/2009	05:22:13.1	N.B.
23/01/2009	05:22:22.9	-0.8
23/01/2009	05:22:35.7	N.B.
23/01/2009	05:22:55.5	N.B.
23/01/2009	05:23:29.2	-2.5
23/01/2009	05:24:00.6	-1.1
23/01/2009	05:24:15.9	N.B.
23/01/2009	05:24:22.8	-1.6
23/01/2009	05:24:31.3	-1.6
23/01/2009	05:24:41.1	N.B.
23/01/2009	05:24:44.5	-1.6
23/01/2009	05:24:50.3	N.B.
23/01/2009	05:25:16.1	-1.1
23/01/2009	05:25:27.2	-1.6
23/01/2009	05:25:30.5	-1.6
23/01/2009	05:25:37.1	-1.6
23/01/2009	05:25:43.2	-1.6
23/01/2009	05:25:50.5	-1.6

23/01/2009	05:26:07.4	-1.6
23/01/2009	05:26:13.4	-1.6
23/01/2009	05:26:48.6	-1.6
23/01/2009	05:26:56.2	-2.5
23/01/2009	05:27:09.3	-1.6
23/01/2009	05:27:11.9	-1.6
23/01/2009	05:27:17.5	-1.6
23/01/2009	05:27:21.8	-2.5
23/01/2009	05:27:31.1	-2.5
23/01/2009	05:27:33.5	-1.6
23/01/2009	05:27:43.7	-1.6
23/01/2009	05:27:47.7	-1.6
23/01/2009	05:27:54.8	-0.8
23/01/2009	05:28:03.2	-1.1
23/01/2009	05:28:13.6	-1.1
23/01/2009	05:28:25.0	-1.6
23/01/2009	05:28:30.6	-1.6
23/01/2009	05:28:35.0	-1.6
23/01/2009	05:28:44.0	-1.6
23/01/2009	05:28:51.9	-1.1
23/01/2009	05:28:59.4	-1.1
23/01/2009	05:29:20.0	-1.1
23/01/2009	05:29:38.5	-1.6
23/01/2009	05:29:48.8	-1.1
23/01/2009	05:29:53.7	-1.6
23/01/2009	05:30:02.4	-1.6
23/01/2009	05:30:08.4	-1.1
23/01/2009	05:30:14.8	-1.6
23/01/2009	05:30:23.9	-1.6
23/01/2009	05:30:27.1	-1.6
23/01/2009	05:30:30.7	-1.6
23/01/2009	05:30:35.2	-1.6
23/01/2009	05:30:46.9	-0.8
23/01/2009	05:30:52.9	-1.6
23/01/2009	05:30:57.3	-1.6
23/01/2009	05:31:01.3	-1.6
23/01/2009	05:31:09.4	-1.1
23/01/2009	05:31:15.5	-1.6
23/01/2009	05:31:18.9	-1.1
23/01/2009	05:31:29.3	-1.6
23/01/2009	05:31:33.9	N.B.
23/01/2009	05:31:36.0	N.B.
23/01/2009	05:31:38.0	-1.1
23/01/2009	05:31:41.8	-1.6
23/01/2009	05:31:50.1	-1.6
23/01/2009	05:31:53.3	-1.6
23/01/2009	05:31:56.4	-2.5
23/01/2009	05:31:58.9	-1.6
23/01/2009	05:32:01.3	-1.6
23/01/2009	05:32:10.6	-1.1
23/01/2009	05:32:16.9	-1.6
23/01/2009	05:32:21.4	-1.6
23/01/2009	05:32:29.0	N.B.
23/01/2009	05:32:30.6	N.B.
23/01/2009	05:32:34.1	-1.6
23/01/2009	05:32:38.5	-1.6
23/01/2009	05:32:41.5	-0.8
23/01/2009	05:32:47.7	-2.5
23/01/2009	05:32:54.7	N.B.
23/01/2009	05:32:56.1	-1.6
23/01/2009	05:32:58.7	-2.5
23/01/2009	05:33:09.4	-1.1
23/01/2009	05:33:14.9	-2.5
23/01/2009	05:33:18.9	-2.5
23/01/2009	05:33:25.2	-1.6
23/01/2009	05:33:27.5	-1.1
23/01/2009	05:33:37.1	-1.1
23/01/2009	05:33:41.9	-1.1
23/01/2009	05:33:47.8	-0.5
23/01/2009	05:33:55.8	-1.6

23/01/2009	05:34:06.7	-1.1
23/01/2009	05:34:11.9	-0.5
23/01/2009	05:34:20.5	-2.5
23/01/2009	05:34:23.4	-1.1
23/01/2009	05:34:30.1	-1.1
23/01/2009	05:34:40.6	-1.6
23/01/2009	05:34:52.2	-1.6
23/01/2009	05:34:56.6	-1.6
23/01/2009	05:35:15.4	-1.6
23/01/2009	05:35:23.6	-1.6
23/01/2009	05:35:26.6	-1.1
23/01/2009	05:35:38.2	-0.8
23/01/2009	05:35:45.8	-0.8
23/01/2009	05:35:53.4	-1.6
23/01/2009	05:35:57.8	-1.6
23/01/2009	05:36:04.1	-1.1
23/01/2009	05:36:13.6	-1.1
23/01/2009	05:36:23.6	-0.8
23/01/2009	05:36:29.8	-0.8
23/01/2009	05:36:37.9	-1.1
23/01/2009	05:36:42.2	-1.6
23/01/2009	05:36:50.7	-1.1
23/01/2009	05:36:55.8	-2.5
23/01/2009	05:36:58.9	-1.1
23/01/2009	05:37:10.7	-0.5
23/01/2009	05:37:17.8	-1.1
23/01/2009	05:37:23.4	-1.6
23/01/2009	05:37:27.4	-1.6
23/01/2009	05:37:29.8	-0.8
23/01/2009	05:37:35.4	-1.6
23/01/2009	05:37:40.1	-1.6
23/01/2009	05:37:47.4	-0.8
23/01/2009	05:37:47.6	-1.1
23/01/2009	05:37:52.9	-1.1
23/01/2009	05:37:56.9	-1.6
23/01/2009	05:38:09.3	-0.8
23/01/2009	05:38:24.6	-1.1
23/01/2009	05:38:35.9	-1.1
23/01/2009	05:38:39.1	-0.8
23/01/2009	05:38:47.7	-1.6
23/01/2009	05:39:04.4	N.B.
23/01/2009	05:39:35.0	-1.1
23/01/2009	05:39:40.1	-1.1
23/01/2009	05:40:14.0	-0.5
23/01/2009	05:40:19.6	-0.5
23/01/2009	05:40:44.0	-1.6
23/01/2009	05:40:50.9	-1.6
23/01/2009	05:40:54.1	-1.6
23/01/2009	05:41:10.5	-1.6
23/01/2009	05:41:16.0	-0.8
23/01/2009	05:42:37.0	0
23/01/2009	05:47:13.3	-1.1
23/01/2009	05:47:41.6	-1.6
23/01/2009	05:48:43.5	-0.5
23/01/2009	05:49:02.3	-1.1
23/01/2009	05:51:36.2	-0.8
15/02/2009	06:02:13.1	0.8
04/05/2009	09:25:20.3	0.2
04/05/2009	09:28:25.7	-0.8
04/05/2009	09:30:11.4	-0.5
04/05/2009	09:31:09.5	0.2
04/05/2009	09:31:27.3	0.2
04/05/2009	09:31:49.0	-0.3
04/05/2009	09:37:19.6	-1.1
04/05/2009	09:37:36.8	-1.1
04/05/2009	09:40:39.0	0.2
04/05/2009	09:43:42.8	-0.8
04/05/2009	09:49:41.5	0.2
23/05/2009	19:30:15.5	-0.8
23/05/2009	19:47:06.0	-0.1

23/05/2009	21:11:36.1	-0.8
25/05/2009	18:01:00.3	0.1
13/06/2009	03:40:44.1	0.9
18/06/2009	00:03:22.1	-0.3
01/07/2009	16:06:50.7	N.B.
03/07/2009	14:20:52.7	0
29/01/2010	17:34:48.3	1.1
29/01/2010	17:38:15.1	0.2
29/01/2010	17:39:05.7	0.9
30/03/2010	16:10:26.7	-1.6
30/03/2010	16:10:29.9	-1.6
30/03/2010	16:10:32.7	-1.6
30/03/2010	16:10:37.2	-0.3
30/03/2010	16:10:51.2	-1.1
30/03/2010	16:11:05.1	-0.5
30/03/2010	16:11:25.1	-0.3
30/03/2010	16:11:47.2	N.D.
30/03/2010	16:11:57.2	-0.8
30/03/2010	16:12:21.5	-0.8
30/03/2010	16:12:54.7	-0.8
30/03/2010	16:12:59.2	-1.6
30/03/2010	16:13:04.4	-0.8
30/03/2010	16:13:16.8	-1.1
30/03/2010	16:13:23.7	-1.1
30/03/2010	16:13:53.8	-0.5
30/03/2010	16:14:07.2	N.D.
30/03/2010	16:14:21.7	-0.8
30/03/2010	16:14:42.6	-1.6
30/03/2010	16:14:46.2	-1.1
30/03/2010	16:14:56.6	N.D.
30/03/2010	16:14:59.9	N.D.
30/03/2010	16:15:06.4	N.D.
30/03/2010	16:15:14.4	-0.8
30/03/2010	16:15:23.2	-0.5
30/03/2010	16:15:29.2	-1.1
30/03/2010	16:15:48.0	-1.1
30/03/2010	16:16:00.5	-0.8
30/03/2010	16:16:12.8	-0.5
30/03/2010	16:16:25.7	-1.1
30/03/2010	16:16:51.7	-0.8
30/03/2010	16:17:15.5	-1.1
30/03/2010	16:17:18.5	N.D.
30/03/2010	16:17:34.9	-0.5
30/03/2010	16:17:54.3	N.D.
30/03/2010	16:18:00.6	-0.5
30/03/2010	16:18:17.3	-0.3
30/03/2010	16:18:32.1	-0.8
30/03/2010	16:18:41.2	N.D.
30/03/2010	16:18:55.4	N.D.
30/03/2010	16:19:02.3	-0.8
30/03/2010	16:19:07.4	N.D.
30/03/2010	16:19:12.3	-0.8
30/03/2010	16:19:19.8	-0.5
30/03/2010	16:19:30.7	N.D.
30/03/2010	16:19:31.6	-1.1
30/03/2010	16:19:34.5	-0.8
30/03/2010	16:19:42.6	N.D.
30/03/2010	16:19:53.3	-0.8
30/03/2010	16:20:01.9	-0.5
30/03/2010	16:20:08.1	-0.8
30/03/2010	16:20:19.2	-1.1
30/03/2010	16:20:28.2	N.D.
30/03/2010	16:20:33.9	N.D.
30/03/2010	16:20:37.0	N.D.
30/03/2010	16:20:43.6	-0.1
30/03/2010	16:21:03.6	N.D.
30/03/2010	16:21:06.1	N.D.
30/03/2010	16:21:36.7	N.D.
30/03/2010	16:21:49.0	-0.5
30/03/2010	16:22:08.8	-0.3

30/03/2010	16:22:31.2	-0.3
30/03/2010	16:22:40.1	-0.3
30/03/2010	16:22:48.3	N.D.
30/03/2010	16:22:56.1	N.D.
30/03/2010	16:22:58.7	-0.8
30/03/2010	16:23:12.1	N.D.
30/03/2010	16:23:26.7	-0.3
30/03/2010	16:23:36.9	-0.5
30/03/2010	16:23:51.0	N.D.
30/03/2010	16:23:55.2	N.D.
30/03/2010	16:24:03.1	0.6
30/03/2010	16:24:18.0	N.D.
30/03/2010	16:24:22.1	N.D.
30/03/2010	16:24:27.0	-0.8
30/03/2010	16:24:36.8	0.9
30/03/2010	16:24:56.9	-0.8
30/03/2010	16:25:08.9	N.D.
30/03/2010	16:25:10.8	N.D.
30/03/2010	16:25:13.2	N.D.
30/03/2010	16:25:19.3	-0.8
30/03/2010	16:25:24.0	N.D.
30/03/2010	16:25:46.3	1
30/03/2010	16:26:10.5	-0.8
30/03/2010	16:26:28.3	-0.8
30/03/2010	16:26:43.4	-0.5
30/03/2010	16:26:52.8	N.D.
30/03/2010	16:27:08.0	-0.1
30/03/2010	16:27:42.4	-0.5
30/03/2010	16:27:47.0	-1.1
30/03/2010	16:28:24.1	0.8
30/03/2010	16:28:48.2	N.D.
30/03/2010	16:28:59.6	N.D.
30/03/2010	16:29:05.9	1.1
30/03/2010	16:29:35.0	N.D.
30/03/2010	16:29:59.4	-0.5
30/03/2010	16:30:17.8	-0.8
30/03/2010	16:30:34.9	0.1
30/03/2010	16:30:43.7	N.D.
30/03/2010	16:30:53.8	1
30/03/2010	16:31:20.0	N.D.
30/03/2010	16:31:31.8	0.6
30/03/2010	16:32:00.0	0.1
30/03/2010	16:32:17.5	-0.1
30/03/2010	16:32:43.6	N.D.
30/03/2010	16:32:45.5	-0.8
30/03/2010	16:32:51.0	0.6
30/03/2010	16:33:19.9	N.D.
30/03/2010	16:33:30.2	-0.5
30/03/2010	16:33:42.9	-0.3
30/03/2010	16:33:54.3	-0.8
30/03/2010	16:34:27.5	1.2
30/03/2010	16:35:01.5	N.D.
30/03/2010	16:35:06.1	-0.1
30/03/2010	16:35:16.1	-0.1
30/03/2010	16:35:42.7	-0.8
30/03/2010	16:35:55.8	-0.5
30/03/2010	16:36:19.5	N.D.
30/03/2010	16:36:23.3	-0.1
30/03/2010	16:36:45.9	0.1
30/03/2010	16:37:08.1	N.D.
30/03/2010	16:37:40.8	-0.3
30/03/2010	16:37:52.6	-0.3
30/03/2010	16:38:21.2	N.D.
30/03/2010	16:38:23.2	0.2
30/03/2010	16:38:49.5	0.2
30/03/2010	16:39:16.6	N.D.
30/03/2010	16:39:31.3	N.D.
30/03/2010	16:39:52.9	N.D.
30/03/2010	16:40:23.6	1.2
30/03/2010	16:43:17.4	-0.8

30/03/2010	16:43:55.2	-0.8
30/03/2010	16:44:15.6	-1.1
30/03/2010	16:44:56.6	-0.1
30/03/2010	16:45:28.8	-0.1
30/03/2010	16:46:47.2	-0.5
30/03/2010	16:48:39.7	-0.8
30/03/2010	16:49:08.0	-0.8
30/03/2010	16:51:15.3	-0.8
30/03/2010	17:16:08.8	0.5
30/03/2010	16:35:29.0	0.1
07/07/2010	13:11:10.7	N.D.
07/07/2010	13:12:00.3	-0.3
07/07/2010	13:16:31.6	N.D.
07/07/2010	13:16:35.8	-0.1
07/07/2010	13:16:45.6	N.D.
07/07/2010	13:16:54.4	-0.3
07/07/2010	13:31:22.9	-0.8
07/07/2010	13:31:30.0	N.D.
07/07/2010	13:32:20.8	N.D.
07/07/2010	16:36:55.9	-0.1
07/07/2010	23:43:33.3	-0.5
20/07/2010	10:12:00.7	-0.3
25/07/2010	13:36:26.7	-0.3
25/07/2010	13:36:34.6	-0.1
25/07/2010	13:36:43.7	-1.1
25/07/2010	13:37:46.2	N.D.
25/07/2010	13:37:54.3	-0.1
25/07/2010	13:38:08.9	-0.8
26/07/2010	19:38:15.5	-1.1
26/07/2010	19:38:28.5	-0.5
26/07/2010	19:40:03.4	-0.5
26/07/2010	19:40:55.8	-1.1
26/07/2010	19:41:01.7	-1.1
26/07/2010	19:41:57.3	-0.5
26/07/2010	19:52:02.8	-0.8
26/07/2010	19:53:40.0	-1.1
27/08/2010	22:37:25.8	-0.1
01/10/2010	07:37:46.9	-0.5
06/11/2010	16:22:01.0	0.8
29/12/2010	20:49:36.3	-0.5
05/02/2011	16:26:31.5	-0.3
07/02/2011	06:26:46.4	-0.8
07/02/2011	06:26:58.1	-1.1
07/02/2011	06:27:09.1	-1.1
07/02/2011	06:27:20.1	N.D.
07/02/2011	06:27:24.5	N.D.
07/02/2011	06:27:35.6	-0.1
07/02/2011	06:28:05.1	-0.8
07/02/2011	06:28:11.1	-0.8
07/02/2011	06:28:19.2	N.D.
07/02/2011	06:28:41.3	-0.5
07/02/2011	06:28:47.3	-0.5
07/02/2011	06:29:04.2	-0.5
07/02/2011	06:29:14.2	N.D.
07/02/2011	06:30:22.6	-0.8
07/02/2011	06:30:31.3	-0.5
07/02/2011	06:30:39.1	N.D.
07/02/2011	06:30:46.6	-1.1
07/02/2011	06:34:27.1	N.D.
07/02/2011	06:34:29.4	N.D.
07/02/2011	06:34:33.7	-0.1
07/02/2011	06:34:46.8	-1.1
07/02/2011	06:34:50.3	-0.8
07/02/2011	06:35:00.3	-0.5
07/02/2011	06:35:53.8	N.D.
07/02/2011	06:36:01.0	0.1
07/02/2011	06:36:09.5	N.D.
07/02/2011	06:45:47.7	-0.5
07/02/2011	06:56:51.0	-0.3
07/02/2011	06:57:02.7	-1.1

07/02/2011	06:58:08.1	-0.5
07/02/2011	07:04:03.0	1.1
07/02/2011	07:07:34.7	-1.1
07/02/2011	07:07:44.8	-1.1
07/02/2011	07:10:30.0	-0.5
07/02/2011	07:10:37.8	-1.6
07/02/2011	07:11:32.8	-1.1
07/02/2011	07:16:58.6	N.D.
07/02/2011	07:17:41.7	-1.6
07/02/2011	07:17:44.3	N.D.
07/02/2011	07:17:46.5	-0.8
07/02/2011	07:21:09.0	-0.5
10/04/2011	12:11:11.7	1.4
18/04/2011	01:44:18.9	-0.3
30/04/2011	00:35:54.2	-0.5
30/04/2011	00:46:02.9	-0.5
10/05/2011	07:48:33.5	-0.8
10/05/2011	07:48:50.3	-0.8
10/05/2011	07:50:34.1	-1.1
10/05/2011	07:51:49.1	-1.1
10/05/2011	08:32:32.7	-0.8
13/05/2011	01:07:22.9	-1.1
13/05/2011	01:07:28.4	-0.8
13/05/2011	01:09:02.0	-1.6
13/05/2011	01:28:21.2	-0.1
13/05/2011	01:28:33.5	-1.1
13/05/2011	02:26:40.9	-0.5
18/05/2011	16:39:12.9	0.7
19/05/2011	00:39:55.7	1.5
19/05/2011	00:43:05.9	-0.5
30/05/2011	03:18:32.9	-0.8
10/07/2011	03:01:59.3	0
10/07/2011	03:02:00.2	0.5
10/07/2011	03:02:00.6	N.D.
23/07/2011	12:27:03.6	-0.1
02/09/2011	19:34:53.2	0.2
08/09/2011	02:56:11.8	1.2
08/09/2011	03:01:27.6	0.7
17/09/2011	00:46:06.0	N.D.
04/10/2011	23:55:40.4	-0.3
28/11/2011	17:40:06.8	0.7
03/12/2011	21:30:48.4	-0.3
14/01/2012	07:42:40.9	0.2
22/01/2012	08:01:05.7	-0.3
25/01/2012	00:23:14.7	-0.3
31/01/2012	18:47:02.0	-0.3
10/02/2012	00:29:09.9	0.1
11/02/2012	03:52:09.5	-0.1
13/02/2012	11:29:33.0	N.D.
25/02/2012	17:47:42.9	-0.5
12/03/2012	16:01:47.1	-0.8
29/03/2012	08:27:58.8	-0.3
11/04/2012	23:07:17.8	-0.3
11/04/2012	23:08:12.0	-0.5
11/04/2012	23:08:22.1	-0.5
11/04/2012	23:15:41.4	-0.8
12/04/2012	21:45:52.3	-0.1
19/04/2012	16:40:30.8	0.1
19/04/2012	16:44:38.2	0.4
19/04/2012	16:45:06.1	-0.8
19/04/2012	16:53:08.2	-0.3
19/04/2012	19:34:01.1	0.8
19/04/2012	19:34:11.0	-0.5
19/04/2012	19:34:47.4	-0.5
19/04/2012	19:56:42.3	1.4
19/04/2012	19:57:22.7	0.2
19/04/2012	19:58:16.4	-0.3
19/04/2012	20:06:03.2	-0.5
19/04/2012	20:12:36.3	-0.8
19/04/2012	22:15:33.7	-0.8

20/04/2012	04:29:05.2	0.8
20/04/2012	04:29:30.0	1.1
20/04/2012	04:30:42.0	-0.8
20/04/2012	04:30:46.1	-0.5
20/04/2012	04:45:53.3	1.1
20/04/2012	05:04:13.8	-0.3
20/04/2012	05:32:31.6	-0.3
22/04/2012	21:37:40.6	0.4
27/05/2012	22:26:38.9	-0.5
27/05/2012	23:05:39.2	-1.1
27/05/2012	23:05:48.2	-1.1
01/06/2012	21:55:28.1	0.1
01/06/2012	22:51:23.6	0.1
05/06/2012	01:55:36.4	0.4
06/07/2012	03:05:48.6	0.2
02/08/2012	13:55:42.6	N.D.
02/08/2012	13:55:44.0	0.2
04/08/2012	01:08:16.5	-0.5
04/08/2012	01:08:23.8	N.D.
04/08/2012	01:09:13.0	-0.1
04/08/2012	01:09:20.0	-0.5
04/08/2012	01:24:57.4	0.1
04/08/2012	17:24:48.6	-0.5
04/08/2012	17:33:31.5	-0.5
04/08/2012	18:35:42.8	-1.1
04/08/2012	21:00:51.3	-0.5
04/08/2012	22:13:31.5	-0.8
05/08/2012	01:07:09.4	-0.5
07/08/2012	02:32:48.1	-1.1
09/08/2012	13:32:54.8	-1.1
09/08/2012	13:46:39.5	-0.8
22/08/2012	07:01:35.0	0.2
26/08/2012	23:14:10.1	0.2
26/08/2012	23:14:19.6	0.2
26/08/2012	23:19:11.7	-0.9
26/08/2012	23:19:14.4	N.D.
26/08/2012	23:19:24.2	0.2
27/08/2012	02:29:12.1	-0.1
31/08/2012	05:53:16.7	-0.1
01/09/2012	03:48:22.8	0.7
01/09/2012	04:02:31.4	1
01/09/2012	04:37:38.4	1
01/09/2012	06:09:23.0	-0.1
07/09/2012	07:15:46.3	0.1
07/09/2012	07:16:25.3	-0.5
07/09/2012	07:16:41.3	-1.1
07/09/2012	07:17:00.0	N.D.
07/09/2012	07:17:04.4	N.D.
07/09/2012	07:17:27.3	N.D.
07/09/2012	07:17:30.8	N.D.
07/09/2012	07:17:46.8	-1.1
07/09/2012	07:17:55.6	-1.1
07/09/2012	07:18:21.3	-0.3
07/09/2012	07:18:47.2	-1.1
07/09/2012	07:18:52.4	-0.5
07/09/2012	07:19:42.8	-1.1
07/09/2012	07:20:07.7	N.D.
07/09/2012	07:20:13.2	N.D.
07/09/2012	07:20:20.0	-0.8
07/09/2012	07:20:30.7	N.D.
07/09/2012	07:20:38.0	-0.5
07/09/2012	07:21:03.5	-0.8
07/09/2012	07:21:10.5	-1.1
07/09/2012	07:21:18.2	N.D.
07/09/2012	07:21:20.1	0.1
07/09/2012	07:21:30.8	N.D.
07/09/2012	07:21:49.1	-0.8
07/09/2012	07:22:09.0	N.D.
07/09/2012	07:22:13.3	N.D.
07/09/2012	07:22:18.1	N.D.

07/09/2012	07:22:30.8	-0.8
07/09/2012	07:22:41.1	-1.1
07/09/2012	07:22:52.2	N.D.
07/09/2012	07:22:54.6	N.D.
07/09/2012	07:23:07.8	N.D.
07/09/2012	07:23:20.8	N.D.
07/09/2012	07:23:34.6	-0.3
07/09/2012	07:23:34.9	N.D.
07/09/2012	07:23:42.2	0.1
07/09/2012	07:23:53.3	-0.8
07/09/2012	07:24:04.5	N.D.
07/09/2012	07:24:09.2	N.D.
07/09/2012	07:24:39.7	0.9
07/09/2012	07:25:03.3	N.D.
07/09/2012	07:25:09.3	N.D.
07/09/2012	07:25:14.9	N.D.
07/09/2012	07:25:35.7	N.D.
07/09/2012	07:25:38.1	N.D.
07/09/2012	07:25:54.3	0.6
07/09/2012	07:26:21.0	N.D.
07/09/2012	07:26:26.3	-0.8
07/09/2012	07:26:37.2	N.D.
07/09/2012	07:26:48.0	N.D.
07/09/2012	07:26:50.0	N.D.
07/09/2012	07:26:52.8	N.D.
07/09/2012	07:26:56.8	N.D.
07/09/2012	07:27:06.9	N.D.
07/09/2012	07:27:26.4	0.9
07/09/2012	07:27:47.9	N.D.
07/09/2012	07:28:16.4	-1.1
07/09/2012	07:29:01.5	N.D.
07/09/2012	07:29:05.6	N.D.
07/09/2012	07:29:28.0	-0.5
07/09/2012	07:29:35.3	-0.5
07/09/2012	07:29:59.9	N.D.
07/09/2012	07:30:49.6	N.D.
07/09/2012	07:30:54.7	N.D.
07/09/2012	07:31:05.8	N.D.
07/09/2012	07:31:17.5	N.D.
07/09/2012	07:31:27.1	0.9
07/09/2012	07:32:57.3	-1.1
07/09/2012	07:33:17.3	0.1
07/09/2012	07:33:42.8	N.D.
07/09/2012	07:33:45.8	0
07/09/2012	07:33:51.8	N.D.
07/09/2012	07:34:14.1	1.7
07/09/2012	07:35:25.4	-0.5
07/09/2012	07:36:09.0	-1.1
07/09/2012	07:37:03.0	0
07/09/2012	07:38:05.4	-0.8
07/09/2012	07:38:33.7	-0.5
07/09/2012	07:38:59.9	-1.1
07/09/2012	07:39:46.8	-0.3
07/09/2012	07:42:36.6	-1.1
07/09/2012	07:42:49.8	-0.8
07/09/2012	07:42:58.3	N.D.
07/09/2012	07:43:14.2	N.D.
07/09/2012	07:43:19.4	-0.8
07/09/2012	07:43:53.3	-0.1
07/09/2012	07:48:13.2	-0.8
07/09/2012	07:48:18.7	N.D.
07/09/2012	07:49:12.1	1.1
07/09/2012	07:50:01.2	N.D.
07/09/2012	07:51:21.3	N.D.
07/09/2012	07:51:26.0	0.1
07/09/2012	07:51:46.8	0.2
07/09/2012	07:52:32.6	N.D.
07/09/2012	07:52:38.8	N.D.
07/09/2012	07:53:17.7	N.D.
07/09/2012	07:53:42.8	-0.8

07/09/2012	07:53:48.6	N.D.
07/09/2012	07:55:27.5	-0.3
07/09/2012	07:55:34.1	0.2
07/09/2012	07:58:46.0	-0.5
07/09/2012	07:59:18.4	0.4
07/09/2012	08:00:46.8	0.5
07/09/2012	08:02:57.0	N.D.
07/09/2012	08:03:02.7	N.D.
07/09/2012	08:03:27.6	1.2
07/09/2012	08:04:40.0	N.D.
07/09/2012	08:04:46.3	N.D.
07/09/2012	08:08:55.3	-0.5
07/09/2012	08:10:17.7	-0.5
07/09/2012	08:11:14.7	N.D.
07/09/2012	08:11:55.9	N.D.
07/09/2012	08:12:08.4	N.D.
07/09/2012	08:12:27.3	N.D.
07/09/2012	08:12:40.5	N.D.
07/09/2012	08:13:19.4	0.9
07/09/2012	08:14:03.0	-0.8
07/09/2012	08:14:12.0	0.2
07/09/2012	08:14:49.8	-0.8
07/09/2012	08:15:18.1	-0.3
07/09/2012	08:15:34.0	N.D.
07/09/2012	08:16:38.0	N.D.
07/09/2012	08:16:49.7	-0.5
07/09/2012	08:17:32.8	0.2
07/09/2012	08:18:30.5	-0.3
07/09/2012	08:18:46.3	-0.5
07/09/2012	08:20:05.8	N.D.
07/09/2012	08:20:16.3	N.D.
07/09/2012	08:20:24.9	N.D.
07/09/2012	08:21:43.4	0
07/09/2012	08:21:48.3	0.8
07/09/2012	08:22:35.4	N.D.
07/09/2012	08:22:44.5	N.D.
07/09/2012	08:24:43.7	-0.5
07/09/2012	08:25:00.9	1.7
07/09/2012	08:25:46.2	-0.8
07/09/2012	08:26:56.1	0.1
07/09/2012	08:28:48.4	-0.5
07/09/2012	08:29:44.3	N.D.
07/09/2012	08:29:49.6	0.5
07/09/2012	08:31:56.9	N.D.
07/09/2012	08:32:05.4	0.8
07/09/2012	08:34:00.8	N.D.
07/09/2012	08:36:03.0	0.6
07/09/2012	08:38:42.5	N.D.
07/09/2012	08:38:51.0	N.D.
07/09/2012	08:39:24.3	-0.8
07/09/2012	08:40:28.9	0.2
07/09/2012	08:41:47.0	0
07/09/2012	08:41:50.6	-0.1
07/09/2012	08:42:20.0	-0.3
07/09/2012	08:42:50.9	-1.1
07/09/2012	08:43:51.8	N.D.
07/09/2012	08:44:06.6	-0.8
07/09/2012	08:47:00.8	1
07/09/2012	08:48:49.3	N.D.
07/09/2012	08:51:21.4	-0.3
07/09/2012	08:52:14.6	-0.8
07/09/2012	08:52:59.3	-0.5
07/09/2012	08:53:23.0	N.D.
07/09/2012	08:54:45.6	-0.1
07/09/2012	08:55:07.9	N.D.
07/09/2012	08:55:47.0	N.D.
07/09/2012	08:55:55.8	-0.5
07/09/2012	08:56:53.6	N.D.
07/09/2012	08:57:57.2	-0.8
07/09/2012	08:58:12.0	-0.8

07/09/2012	08:59:11.6	N.D.
07/09/2012	09:02:16.1	0.1
07/09/2012	09:02:39.3	N.D.
07/09/2012	09:03:03.4	N.D.
07/09/2012	09:03:13.0	N.D.
07/09/2012	09:04:19.2	N.D.
07/09/2012	09:04:23.8	N.D.
07/09/2012	09:04:36.5	-0.5
07/09/2012	09:06:45.6	0
07/09/2012	09:06:56.9	-0.1
07/09/2012	09:11:39.6	N.D.
07/09/2012	09:12:26.3	N.D.
07/09/2012	09:12:48.2	N.D.
07/09/2012	09:13:43.9	-0.3
07/09/2012	09:14:34.1	N.D.
07/09/2012	09:20:54.7	N.D.
07/09/2012	09:22:50.8	N.D.
07/09/2012	09:24:21.6	-0.1
07/09/2012	09:35:05.6	N.D.
07/09/2012	09:35:15.9	N.D.
07/09/2012	11:00:13.4	N.D.
15/09/2012	09:01:17.0	-1.6
15/09/2012	09:02:22.4	-0.8
15/09/2012	09:02:42.0	-1.6
15/09/2012	09:03:48.4	-0.8
15/09/2012	09:03:56.2	-1.6
15/09/2012	09:14:54.7	-0.8
15/09/2012	09:18:10.9	-0.5
15/09/2012	09:22:08.9	-1.6
15/09/2012	09:22:14.5	-1.6
15/09/2012	09:22:18.3	-1.6
15/09/2012	09:22:23.1	-1.1
15/09/2012	09:22:29.2	-0.5
15/09/2012	09:28:05.9	-1.1
15/09/2012	09:28:11.5	-1.6
15/09/2012	09:28:20.4	-1.6
15/09/2012	09:28:23.8	-0.5
15/09/2012	09:47:06.9	-0.3
15/09/2012	09:50:37.3	-0.5
15/09/2012	09:54:06.7	-0.3
15/09/2012	09:57:48.1	-1.6
15/09/2012	09:57:50.4	N.D.
15/09/2012	09:57:52.1	-0.8
15/09/2012	10:02:13.3	-1.6
15/09/2012	10:02:36.3	-1.1
15/09/2012	10:12:29.9	-1.6
21/09/2012	09:26:20.2	0.7
03/10/2012	00:24:21.9	-1.1
03/10/2012	00:25:28.6	0.1
03/10/2012	00:28:14.3	-0.8
03/10/2012	00:29:29.4	-0.1
03/10/2012	00:38:25.9	-0.8
04/10/2012	23:43:44.4	0.1
11/10/2012	22:11:53.1	-0.1
14/10/2012	15:22:29.9	0.2
16/10/2012	00:05:40.4	-1.1
16/10/2012	00:05:52.9	1.8
27/10/2012	03:09:46.5	0.5
27/10/2012	04:25:56.0	-0.5
27/10/2012	07:33:34.4	0.2
13/12/2012	05:00:24.6	-0.5
13/12/2012	05:05:17.1	0
16/12/2012	06:50:59.9	-0.5
22/12/2012	23:55:54.4	-0.3
23/12/2012	14:38:29.4	-0.3
23/12/2012	14:47:19.7	-0.5
23/12/2012	16:55:34.8	-1.1
23/12/2012	16:56:30.1	-0.3
23/12/2012	16:57:10.4	-0.8
24/12/2012	08:08:07.9	0.2

25/12/2012	18:10:28.3	0.4
26/12/2012	06:30:16.0	N.D.
26/12/2012	06:30:16.9	-0.8
26/12/2012	06:30:42.4	-0.5
26/12/2012	06:31:12.6	-0.5
26/12/2012	06:33:01.8	0.1
26/12/2012	06:33:13.0	-1.1
26/12/2012	06:33:21.2	-0.8
26/12/2012	06:34:23.1	-0.5
27/12/2012	21:16:05.4	0.4
29/12/2012	19:27:33.8	0.1
31/12/2012	00:26:57.0	-0.5
31/12/2012	00:27:22.5	-0.3
31/12/2012	00:36:52.1	0.2
31/12/2012	00:58:27.8	-0.3
02/01/2013	16:17:55.6	-0.3
02/01/2013	16:18:40.1	-0.3
02/01/2013	16:18:58.2	-0.5
02/01/2013	18:28:36.6	-0.8
02/01/2013	18:28:48.9	-0.8
02/01/2013	18:29:17.8	-1.1
05/01/2013	20:44:12.1	0.1
15/01/2013	19:04:44.6	-0.8
18/01/2013	23:50:21.1	-0.5
20/01/2013	04:07:26.1	-0.8
20/01/2013	04:07:37.9	-1.1
23/01/2013	23:44:27.7	-0.1
26/01/2013	09:33:07.7	-0.1
26/01/2013	10:41:24.3	-0.8
26/01/2013	10:42:34.8	0.2
26/01/2013	12:11:23.3	-0.8
26/01/2013	12:12:36.6	-0.5
30/01/2013	23:05:32.0	0
30/01/2013	23:05:34.3	0.2
31/01/2013	23:23:20.7	-0.5
04/02/2013	22:41:33.4	-0.8
07/02/2013	00:14:26.3	-0.3
15/02/2013	10:11:29.2	-0.8
30/03/2013	10:21:35.1	-0.8
03/04/2013	02:09:36.5	-1.1
03/04/2013	02:09:50.9	-0.3
04/04/2013	15:43:20.4	N.D.
05/04/2013	21:55:34.8	-0.8
05/04/2013	21:55:45.1	-0.1
09/04/2013	03:55:18.3	-0.3
12/05/2013	02:41:48.1	-1.1
12/05/2013	02:41:59.5	-0.5
12/05/2013	02:42:06.9	-0.8
12/05/2013	02:48:36.3	-0.8
12/05/2013	02:49:02.9	-1.1
12/05/2013	02:49:14.0	-1.1
12/05/2013	02:49:40.2	-1.6
12/05/2013	02:49:53.4	-1.6
12/05/2013	02:50:08.9	-0.8
12/05/2013	02:50:38.4	-1.1
12/05/2013	02:50:53.5	-1.1
12/05/2013	02:51:11.0	-0.8
12/05/2013	02:51:23.0	-1.1
12/05/2013	02:51:48.9	-0.8
12/05/2013	02:52:15.1	-1.1
12/05/2013	02:52:30.5	-1.1
12/05/2013	02:53:33.7	-1.1
22/12/2013	00:28:35.3	0.9
15/02/2014	06:00:14.3	0.1
20/03/2014	04:36:23.3	0.9
23/03/2014	22:20:32.1	-0.1
31/03/2014	22:59:37.8	-1.1
31/03/2014	22:59:41.9	-1.1
31/03/2014	23:00:14.0	-1.1
31/03/2014	23:01:27.2	-1.1

31/03/2014	23:01:30.7	-1.6
31/03/2014	23:01:34.9	-1.6
31/03/2014	23:01:39.1	-1.6
31/03/2014	23:01:44.6	-0.8
31/03/2014	23:02:05.0	N.D.
31/03/2014	23:02:06.6	N.D.
31/03/2014	23:02:07.8	N.D.
31/03/2014	23:02:10.3	N.D.
31/03/2014	23:02:12.3	N.D.
31/03/2014	23:02:13.9	N.D.
31/03/2014	23:02:19.6	-0.3
31/03/2014	23:02:25.5	-0.8
31/03/2014	23:02:31.6	-0.8
31/03/2014	23:02:36.5	N.D.
31/03/2014	23:02:45.3	-1.1
31/03/2014	23:03:06.3	-0.8
31/03/2014	23:03:17.0	-1.1
31/03/2014	23:03:35.0	N.D.
31/03/2014	23:03:43.1	-0.8
31/03/2014	23:03:51.6	-0.8
31/03/2014	23:04:03.0	N.D.
31/03/2014	23:04:07.5	N.D.
31/03/2014	23:04:09.5	N.D.
31/03/2014	23:04:20.2	-1.1
31/03/2014	23:04:51.8	-0.5
31/03/2014	23:05:34.2	-1.1
31/03/2014	23:05:49.4	N.D.
31/03/2014	23:05:50.8	-0.8
31/03/2014	23:06:08.5	N.D.
31/03/2014	23:06:23.6	-1.6
31/03/2014	23:06:38.2	-1.1
31/03/2014	23:07:00.0	N.D.
31/03/2014	23:07:04.8	-0.8
31/03/2014	23:07:32.8	N.D.
31/03/2014	23:07:35.6	-0.1
31/03/2014	23:08:00.9	-0.1
31/03/2014	23:09:05.3	-1.1
31/03/2014	23:09:36.9	N.D.
31/03/2014	23:09:40.3	N.D.
31/03/2014	23:09:50.8	N.D.
31/03/2014	23:09:52.7	-1.1
31/03/2014	23:10:08.7	-0.5
31/03/2014	23:10:21.6	N.D.
31/03/2014	23:10:23.7	N.D.
31/03/2014	23:11:30.2	-1.1
31/03/2014	23:11:40.4	N.D.
31/03/2014	23:12:15.2	N.D.
31/03/2014	23:12:31.5	N.D.
31/03/2014	23:12:36.6	N.D.
31/03/2014	23:12:43.8	-1.6
31/03/2014	23:13:00.0	N.D.
31/03/2014	23:13:29.1	N.D.
31/03/2014	23:13:43.2	-1.1
31/03/2014	23:13:49.4	-1.1
31/03/2014	23:14:13.4	N.D.
31/03/2014	23:14:27.8	-1.1
31/03/2014	23:15:27.0	N.D.
31/03/2014	23:16:58.4	-0.8
31/03/2014	23:21:41.5	-1.6
31/03/2014	23:22:07.4	0.7
31/03/2014	23:23:38.2	N.D.
06/04/2014	05:10:27.9	0.1
06/04/2014	05:10:43.4	-0.8
06/04/2014	05:31:45.7	-0.1
06/04/2014	05:31:52.9	0.1
06/04/2014	05:32:23.7	-0.5
13/06/2014	20:02:01.1	0.5
25/06/2014	17:30:55.8	-0.1
26/06/2014	09:38:18.6	0.4
30/07/2014	16:30:58.5	-0.8

30/07/2014	16:31:11.2	-1.1
30/07/2014	16:51:29.6	-1.6
30/07/2014	16:51:38.2	-0.5
30/07/2014	16:54:59.0	-1.1
30/07/2014	16:59:26.7	-0.8
30/07/2014	20:53:38.4	-1.6
31/07/2014	11:20:11.4	-0.8
31/07/2014	11:20:22.4	-1.6
31/07/2014	16:13:35.8	-0.8
01/08/2014	16:06:17.5	0.4
01/08/2014	16:53:41.7	0.2
01/08/2014	16:54:29.0	0.1
01/08/2014	22:32:23.8	-0.3
02/08/2014	11:25:35.7	-0.8
02/08/2014	18:06:20.6	0.4
03/08/2014	15:16:38.1	-0.5
03/08/2014	15:53:18.2	-0.5
03/08/2014	16:00:27.1	0.8
03/08/2014	16:00:42.8	-0.5
03/08/2014	16:00:47.5	0.7
04/08/2014	01:35:13.0	-0.3
04/08/2014	01:36:18.2	-0.5
04/08/2014	01:39:32.9	-1.1
07/08/2014	13:48:03.4	-0.5
07/08/2014	13:48:31.8	0.2
07/08/2014	13:48:40.0	-0.3
07/08/2014	15:41:56.5	0.6
07/08/2014	23:57:20.5	0.2
07/08/2014	23:57:25.3	-0.1
07/08/2014	23:57:34.3	-0.1
08/08/2014	00:08:12.9	0.2
08/08/2014	04:47:51.6	-0.1
08/08/2014	04:50:42.4	-0.1
08/08/2014	07:56:34.2	0.2
10/08/2014	13:09:48.4	0.4
10/08/2014	14:24:02.3	0.6
10/08/2014	16:01:04.1	0.2
12/08/2014	22:12:54.5	0.2
12/08/2014	22:13:02.1	-1.1
12/08/2014	22:13:06.9	-1.1
12/08/2014	22:13:12.4	N.D.
12/08/2014	22:18:44.1	0.4
30/08/2014	15:51:50.1	N.D.
30/08/2014	15:51:51.1	N.D.
30/08/2014	15:51:51.7	0.1
30/08/2014	15:52:01.0	-0.5
30/08/2014	22:12:02.0	-0.1
30/08/2014	22:15:17.5	-0.1
03/09/2014	12:14:31.9	-0.3
03/09/2014	12:14:42.3	-1.1
04/09/2014	18:54:37.1	0.9
04/09/2014	18:55:03.8	1
04/09/2014	19:55:32.2	0.9
04/09/2014	20:44:21.4	0.7
04/09/2014	20:44:45.8	-0.3
05/09/2014	14:30:51.4	0.1
10/09/2014	03:09:00.8	N.D.
10/09/2014	03:09:29.6	0.2
10/09/2014	13:59:34.7	-0.5
16/09/2014	01:01:47.2	-0.8
16/09/2014	01:05:44.5	-1.1
16/09/2014	01:05:57.6	-0.8
16/09/2014	01:06:33.9	0.5
16/09/2014	01:21:19.8	1
16/09/2014	01:34:01.4	0.2
16/09/2014	01:45:12.9	1
16/09/2014	04:17:37.9	0.1
16/09/2014	08:30:11.0	0.4
16/09/2014	19:56:17.8	0.4
16/09/2014	21:09:31.2	0.2

16/09/2014	21:11:04.6	0.1
17/09/2014	03:31:18.8	-0.3
19/09/2014	19:48:30.9	0.9
20/09/2014	22:08:06.2	-0.1
20/09/2014	22:08:09.1	N.D.
20/09/2014	22:08:15.6	N.D.
20/09/2014	22:09:41.5	0.6
20/09/2014	22:09:48.2	0.7
20/09/2014	22:09:56.2	0.1
20/09/2014	22:10:05.2	-0.8
21/09/2014	07:30:04.1	-0.3
21/09/2014	07:32:38.7	0.1
21/09/2014	07:32:56.5	-0.3
21/09/2014	07:34:50.5	-0.8
21/09/2014	16:19:57.0	-0.3
25/09/2014	04:20:57.8	N.D.
25/09/2014	04:21:22.0	N.D.
25/09/2014	04:21:25.1	0
25/09/2014	04:21:26.4	-0.1
26/09/2014	10:45:10.5	-0.1
26/09/2014	10:45:41.8	N.D.
28/09/2014	02:47:28.9	0.1
28/09/2014	03:39:40.3	-0.3
28/09/2014	11:25:47.3	-0.1
04/10/2014	06:22:45.5	-0.1
04/10/2014	06:55:30.9	-0.3
12/10/2014	01:53:41.7	0.1
23/10/2014	10:24:06.0	-0.5
25/10/2014	19:29:27.4	N.D.
25/10/2014	19:29:29.0	0.5
25/10/2014	19:29:35.0	N.D.
25/10/2014	19:30:05.1	-0.3
25/10/2014	19:30:08.4	-0.8
25/10/2014	19:30:12.1	-0.3
25/10/2014	19:30:24.4	-0.8
25/10/2014	19:30:51.7	-0.8
25/10/2014	19:31:00.0	-0.5
25/10/2014	19:31:00.0	N.D.
25/10/2014	19:31:08.4	-1.1
25/10/2014	19:32:56.0	-0.8
25/10/2014	22:34:35.0	0.4
30/10/2014	12:53:59.2	0.1
30/10/2014	12:54:47.1	N.D.
30/10/2014	12:54:51.0	1.9
21/11/2014	17:46:59.6	-0.3
21/11/2014	17:47:18.7	-0.8
21/11/2014	17:47:41.0	-0.8
21/11/2014	17:47:47.4	-1.1
21/11/2014	17:48:35.9	N.D.
21/11/2014	17:48:47.9	0.2
22/11/2014	11:52:25.4	-0.5
02/02/2015	21:59:27.7	0.4
02/02/2015	22:01:06.7	0.9
25/02/2015	21:08:55.9	-0.8
25/02/2015	21:09:16.1	-1.6
25/02/2015	21:09:28.4	-1.1
25/02/2015	21:10:10.3	-0.1
25/02/2015	21:10:18.7	-1.1
02/03/2015	19:45:49.9	-0.3
02/03/2015	19:46:08.1	-0.5
02/03/2015	19:48:02.4	-0.3
02/03/2015	19:48:12.9	0.7
02/03/2015	21:17:34.6	0.2
03/03/2015	22:10:56.6	-0.1
03/03/2015	22:31:26.3	-1.1
03/03/2015	22:36:26.8	-0.8
03/03/2015	22:36:31.0	0.4
03/03/2015	22:40:14.5	-0.5
03/03/2015	22:42:29.4	-1.1
03/03/2015	22:42:59.1	-0.1

03/03/2015	22:43:18.7	-1.1
03/03/2015	22:44:14.2	-0.5
03/03/2015	22:44:27.0	-1.1
03/03/2015	22:46:25.1	-1.1
03/03/2015	22:47:24.8	-0.5
03/03/2015	22:49:47.7	1.8
03/03/2015	22:59:40.8	-0.8
03/03/2015	23:09:19.6	-0.8
07/03/2015	11:31:49.7	-1.1
07/03/2015	11:32:55.9	-1.1
07/03/2015	13:32:50.5	-0.3
13/03/2015	03:16:59.6	-1.1
13/03/2015	03:21:11.9	N.D.
13/03/2015	03:21:14.7	-0.5
16/03/2015	04:42:23.0	-0.5
16/03/2015	04:42:39.6	-1.1
21/03/2015	23:28:55.0	0.2
25/03/2015	00:48:10.0	-0.3
27/03/2015	04:37:47.3	N.D.
27/03/2015	04:37:52.4	N.D.
27/03/2015	04:38:05.4	N.D.
27/03/2015	04:38:26.6	-0.5
27/03/2015	07:22:48.6	-0.5
27/03/2015	07:23:03.7	-0.3
27/03/2015	07:31:18.7	-0.3
27/03/2015	07:31:27.8	-1.1
27/03/2015	07:31:37.8	-0.5
20/04/2015	23:06:21.3	0.5
25/04/2015	18:25:41.5	-0.3
03/05/2015	04:06:02.6	-0.8
03/05/2015	04:06:07.1	-1.6
03/05/2015	04:07:40.2	-0.5
09/05/2015	05:53:19.9	-0.1
09/05/2015	08:31:26.8	-0.5
20/05/2015	03:43:03.9	-0.1
21/05/2015	03:26:38.3	-0.1
02/06/2015	17:17:06.0	-0.5
02/06/2015	17:17:33.0	-0.5
02/06/2015	17:20:50.3	-0.5
02/06/2015	17:22:14.3	-0.3
02/06/2015	19:47:45.2	-0.3
06/06/2015	10:49:35.2	-0.1
06/06/2015	19:10:05.4	0.4
11/07/2015	15:35:11.5	0.6
11/07/2015	15:36:03.5	-0.3
11/07/2015	15:36:37.8	0.4
11/07/2015	15:44:24.0	-0.3
14/07/2015	12:16:06.4	0.1
14/07/2015	12:16:54.3	-0.3
15/07/2015	00:28:18.3	-0.8
15/07/2015	00:28:21.5	-1.1
15/07/2015	00:28:24.6	-1.1
15/07/2015	00:28:38.5	-0.5
15/07/2015	00:28:48.2	-1.6
16/07/2015	20:30:16.6	-1.1
22/07/2015	05:38:30.4	-0.1
11/08/2015	00:00:40.1	0.4
31/08/2015	16:43:09.2	0.4
02/09/2015	01:17:28.7	-0.5
02/09/2015	01:17:33.5	-0.5
02/09/2015	01:17:53.8	-0.8
02/09/2015	01:19:08.0	0.1
02/09/2015	01:19:34.3	-1.1
02/09/2015	01:52:44.3	-0.5
05/09/2015	13:03:40.8	-0.8
05/09/2015	13:03:50.8	-0.5
08/09/2015	20:28:18.4	0.1
09/09/2015	02:14:09.8	-0.1
09/09/2015	02:27:20.2	-0.8
10/09/2015	20:30:44.3	-0.1

18/09/2015	02:37:34.8	0.2
18/09/2015	02:48:22.9	-0.3
19/09/2015	03:21:34.4	N.D.
19/09/2015	03:21:35.0	-0.1
19/09/2015	03:22:21.9	-0.5
19/09/2015	03:22:28.7	-0.8
19/09/2015	03:22:34.5	-1.6
19/09/2015	03:22:37.5	-0.5
19/09/2015	03:27:37.5	0.4
27/09/2015	03:55:20.8	0.1
07/10/2015	07:20:34.3	1.5
07/10/2015	07:20:39.8	-0.8
07/10/2015	07:20:55.1	0.5
07/10/2015	07:21:16.6	0.2
07/10/2015	07:24:25.4	-0.1
07/10/2015	07:24:55.1	1.1
07/10/2015	07:26:16.3	-1.1
07/10/2015	07:26:18.8	1
07/10/2015	07:31:26.5	-0.5
07/10/2015	07:31:33.1	1.9
07/10/2015	07:35:03.6	-0.8
07/10/2015	07:36:04.2	-0.5
07/10/2015	07:37:40.7	2.2
07/10/2015	07:39:43.3	-0.8
07/10/2015	07:45:30.7	-0.5
07/10/2015	07:45:43.0	0.6
07/10/2015	07:49:29.1	-0.3
07/10/2015	07:54:06.0	-1.1
07/10/2015	07:54:13.5	2
07/10/2015	07:58:06.9	-0.8
07/10/2015	07:59:08.4	0.7
07/10/2015	07:59:15.9	1.1
07/10/2015	08:02:34.4	0.2
07/10/2015	08:02:52.1	1
07/10/2015	08:03:50.9	-0.5
07/10/2015	08:04:03.1	-0.8
07/10/2015	08:13:51.2	2.3
07/10/2015	08:16:22.4	-0.8
07/10/2015	08:46:36.8	-0.3
07/10/2015	08:49:08.3	1.1
07/10/2015	09:01:37.0	-0.8
07/10/2015	09:10:50.7	2.5
07/10/2015	09:16:24.2	1.1
07/10/2015	11:53:35.6	-0.5
07/10/2015	22:05:20.6	0.2
08/10/2015	13:42:17.9	0.8
10/10/2015	04:00:22.3	0.4
10/10/2015	04:01:57.1	-0.3
10/10/2015	04:02:13.0	-0.8
10/10/2015	04:02:18.7	N.D.
10/10/2015	04:02:40.1	0.4
10/10/2015	04:03:26.7	-0.3
29/10/2015	22:37:16.0	0.7
29/10/2015	22:49:03.9	1.1
29/10/2015	22:49:51.4	0.6
29/10/2015	22:49:56.4	0.4
29/10/2015	22:50:41.1	-0.8
29/10/2015	22:50:53.7	N.D.
29/10/2015	22:50:58.1	-0.3
29/10/2015	22:51:15.7	0.7
29/10/2015	22:55:53.7	0.6
29/10/2015	23:10:12.5	-0.8
29/10/2015	23:10:19.5	0.1
29/10/2015	23:32:19.0	-0.8
30/10/2015	01:00:16.9	N.D.
30/10/2015	01:00:20.1	-0.8
30/10/2015	01:03:55.8	-0.8
30/10/2015	01:14:41.4	-0.5
30/10/2015	01:14:46.1	0.4
30/10/2015	01:15:14.0	0.4

30/10/2015	01:15:30.4	-0.8
30/10/2015	21:38:14.9	-0.5
02/12/2015	18:01:39.8	-0.5
03/12/2015	02:24:26.2	0.2
03/12/2015	02:25:41.3	-0.8
03/12/2015	16:35:50.2	-0.1
12/12/2015	14:03:01.1	-0.8
12/12/2015	14:03:11.8	-1.6
12/12/2015	14:03:45.6	0.8
12/12/2015	14:04:18.5	0.2
12/12/2015	14:05:41.7	-1.1
21/12/2015	01:37:06.6	-0.5
21/12/2015	01:41:04.0	0.5
23/12/2015	18:17:59.1	-0.5
27/12/2015	16:48:26.5	-1.1
27/12/2015	18:06:52.8	-0.5
27/12/2015	19:01:20.9	-0.8
27/12/2015	19:11:35.0	-1.1
27/12/2015	19:24:35.9	-1.1
27/12/2015	19:25:34.7	-1.6
27/12/2015	19:25:46.7	-0.3
27/12/2015	19:25:53.5	-0.1
27/12/2015	19:26:14.1	-1.1
27/12/2015	19:26:17.1	-1.1
17/01/2016	05:23:08.4	-0.3
17/01/2016	05:23:17.2	0.4
17/01/2016	05:23:27.6	0.2
17/01/2016	05:23:50.0	0.2
26/01/2016	04:55:28.5	0.7
05/02/2016	13:19:03.0	0.5
06/02/2016	16:42:56.6	-0.3
10/02/2016	04:20:25.9	0.4
10/02/2016	04:21:15.5	-0.3
02/03/2016	15:45:35.7	-0.5
21/03/2016	08:42:33.7	-1.1
21/03/2016	17:16:04.8	0.1
21/03/2016	23:05:54.4	0.2
24/03/2016	10:59:36.7	0.5
24/03/2016	10:59:51.4	0.5
24/03/2016	11:03:04.9	0.6
29/03/2016	12:57:04.5	-0.3
04/04/2016	09:04:41.2	-1.1
04/04/2016	16:39:11.7	-0.8
08/04/2016	16:34:03.8	-0.5
08/04/2016	16:43:47.7	-0.5
08/04/2016	17:13:00.6	-0.8
09/04/2016	04:18:45.6	-0.3
09/04/2016	07:51:34.4	0.1
20/04/2016	08:42:16.2	-1.1
20/04/2016	08:42:24.6	-0.3
20/04/2016	08:44:04.6	-1.1
20/04/2016	08:44:23.0	-0.3
26/04/2016	02:09:38.2	N.D.
26/04/2016	02:09:38.9	-1.1
26/04/2016	02:09:42.0	-0.3
26/04/2016	02:10:07.2	0.1
26/04/2016	04:42:45.3	-0.3
28/04/2016	23:15:29.6	-1.1
28/04/2016	23:20:09.9	-0.8
28/04/2016	23:20:14.1	-1.1
28/04/2016	23:20:24.3	-1.6
28/04/2016	23:20:47.5	-1.1
28/04/2016	23:20:52.0	-0.3
28/04/2016	23:21:19.6	-1.6
28/04/2016	23:21:22.1	-0.8
28/04/2016	23:21:33.0	-0.8
28/04/2016	23:22:11.1	-1.6
28/04/2016	23:22:20.0	-1.6
28/04/2016	23:22:24.4	-1.6
28/04/2016	23:22:30.2	-1.1

28/04/2016	23:22:42.5	-1.1
28/04/2016	23:43:05.9	-0.8
02/05/2016	12:24:34.8	-0.8
02/05/2016	12:24:40.4	-0.8
02/05/2016	12:24:47.6	-0.8
02/05/2016	12:25:54.2	-0.8
02/05/2016	12:26:05.0	-0.5
02/05/2016	12:49:42.0	-0.1
03/05/2016	21:14:25.5	0.6
04/05/2016	13:02:12.3	-0.5
04/05/2016	13:06:11.8	-0.8
04/05/2016	18:48:21.5	-1.1
05/05/2016	03:32:08.6	-0.8
05/05/2016	03:33:28.1	-0.1
05/05/2016	05:53:35.0	-1.1
06/05/2016	01:47:39.2	-0.8
06/05/2016	01:47:44.8	-1.1
06/05/2016	01:48:24.6	-1.1
06/05/2016	01:48:27.1	-1.1
06/05/2016	01:51:09.0	-1.1
06/05/2016	06:14:03.5	-1.1
06/05/2016	19:33:45.1	-0.3
07/05/2016	06:56:37.7	-0.1
18/05/2016	00:11:46.7	0.6
18/05/2016	23:07:38.0	-0.5
18/05/2016	23:08:49.2	-1.1
18/05/2016	23:09:21.4	0.1
18/05/2016	23:09:33.9	-0.8
18/05/2016	23:16:28.0	-0.3
21/05/2016	08:44:56.9	-0.1
22/05/2016	01:24:24.1	-0.8
24/05/2016	00:33:22.9	-0.1
24/05/2016	00:33:36.6	-0.1
24/05/2016	00:43:36.6	0.2
27/05/2016	08:58:17.8	-0.5
27/05/2016	08:59:29.3	-1.1
27/05/2016	11:04:16.4	-0.8
27/05/2016	13:41:40.6	-0.8
27/05/2016	15:39:18.0	-0.3
27/05/2016	15:44:45.0	-0.1
30/05/2016	20:40:05.1	-0.3
03/06/2016	19:08:49.2	-0.1
03/06/2016	19:09:46.2	0.4
03/06/2016	19:13:11.4	-0.8
03/06/2016	21:44:17.5	0.8
03/06/2016	22:50:06.5	0.1
04/06/2016	02:18:59.8	-0.1
04/06/2016	02:38:53.0	1.5
04/06/2016	02:45:58.8	1.6
04/06/2016	03:27:35.0	-0.8
04/06/2016	03:50:32.9	0.1
04/06/2016	04:26:13.7	0.2
04/06/2016	14:14:03.9	-0.1
10/06/2016	08:32:32.2	0.5
14/06/2016	12:36:59.7	-0.3
20/06/2016	21:08:52.2	-1.1
24/06/2016	21:53:29.8	N.D.
24/06/2016	21:53:32.8	-0.1
24/06/2016	21:53:41.2	N.D.
24/06/2016	21:53:43.0	-0.3
24/06/2016	21:53:52.8	-1.1
27/06/2016	19:58:17.4	-0.5
30/06/2016	18:43:57.4	-0.8
30/06/2016	18:44:05.2	-1.1
30/06/2016	18:44:10.6	N.D.
30/06/2016	18:44:11.8	N.D.
30/06/2016	18:44:16.1	N.D.
30/06/2016	18:44:17.2	-0.3
30/06/2016	18:46:02.7	-0.8
03/07/2016	00:06:45.0	-0.8

03/07/2016	00:06:57.4	-1.1
03/07/2016	00:07:31.3	N.D.
03/07/2016	00:23:41.8	0.1
03/07/2016	00:24:28.7	N.D.
03/07/2016	00:24:29.8	N.D.
03/07/2016	00:24:37.0	-0.8
03/07/2016	00:24:48.6	-0.8
03/07/2016	00:25:51.1	0.1
03/07/2016	00:26:50.8	-1.1
03/07/2016	00:30:08.8	-0.3
03/07/2016	00:36:54.8	-1.1
03/07/2016	00:54:30.0	-1.1
03/07/2016	00:54:36.4	-0.8
03/07/2016	02:34:12.9	0.8
03/07/2016	02:38:31.5	-1.6
03/07/2016	02:38:40.4	-0.8
03/07/2016	02:38:47.5	-0.8
03/07/2016	02:42:54.8	0.1
03/07/2016	02:43:25.5	-1.1
03/07/2016	02:43:32.2	-1.6
03/07/2016	02:44:38.1	-0.8
03/07/2016	02:45:00.2	-0.1
03/07/2016	02:45:20.7	N.D.
03/07/2016	02:45:22.0	-1.1
03/07/2016	02:45:34.8	-0.8
03/07/2016	02:45:39.0	-1.6
03/07/2016	02:45:47.7	-1.1
03/07/2016	02:45:55.4	-1.6
03/07/2016	02:46:27.0	0.7
03/07/2016	02:47:59.2	-1.1
03/07/2016	02:48:02.5	-0.8
03/07/2016	02:48:29.8	-1.1
03/07/2016	02:50:32.2	N.D.
03/07/2016	02:50:32.6	N.D.
03/07/2016	02:50:37.0	-1.1
03/07/2016	02:51:03.1	-1.1
03/07/2016	02:51:30.9	N.D.
03/07/2016	02:51:31.7	-1.1
03/07/2016	03:12:04.2	0.2
03/07/2016	03:12:42.5	-1.6
03/07/2016	03:20:28.2	N.D.
03/07/2016	03:20:28.7	-1.1
03/07/2016	03:56:32.0	-1.1
03/07/2016	05:12:32.0	-1.1
03/07/2016	05:20:41.7	0.2
03/07/2016	10:40:51.3	-0.5
05/07/2016	01:22:39.7	2.1
05/07/2016	02:33:02.7	0.5
05/07/2016	13:50:33.7	-0.1
05/07/2016	21:21:21.2	-0.8
05/07/2016	21:21:59.1	-1.1
05/07/2016	21:22:04.4	-0.8
05/07/2016	21:22:10.4	-0.8
05/07/2016	21:24:49.9	-0.3
06/07/2016	12:19:05.5	0.2
06/07/2016	12:42:52.1	-1.1
06/07/2016	12:42:57.3	-0.8
06/07/2016	12:43:01.8	-0.8
06/07/2016	13:25:00.7	0.6
06/07/2016	22:04:43.0	-0.5
07/07/2016	02:14:16.2	N.D.
07/07/2016	02:14:18.8	-0.1
08/07/2016	20:49:16.8	-0.3
12/07/2016	04:27:48.2	0.1
13/07/2016	02:51:36.3	N.D.
13/07/2016	12:25:17.1	-0.1
14/07/2016	00:39:01.5	-0.3
15/07/2016	15:24:35.8	0.2
18/07/2016	08:16:44.1	-0.8
18/07/2016	08:37:10.0	-0.3

21/07/2016	07:20:50.4	-1.6
21/07/2016	07:21:13.4	-1.1
21/07/2016	07:21:23.1	-0.3
21/07/2016	07:21:50.5	-1.6
21/07/2016	07:22:09.8	-1.1
21/07/2016	07:22:21.3	-1.1
21/07/2016	07:22:24.2	-0.3
21/07/2016	07:24:19.1	-1.6
21/07/2016	07:24:40.9	-0.8
21/07/2016	07:25:20.9	-1.6
21/07/2016	07:25:32.1	-1.1
23/07/2016	05:01:48.6	-0.3
23/07/2016	05:14:36.2	-0.1
26/07/2016	07:35:00.4	-1.1
28/07/2016	16:37:04.6	N.D.
28/07/2016	16:37:07.7	0.7
31/07/2016	22:38:41.9	-0.5

Supplementary dataset 2: Vertical displacement at RITE CGPS 2000_2016

INGV-OV GPS Station Weekly Position (UP) Time Series
ID station: RITE; XYZ position (meters): 4687357.028 1179665.364 4147662.258

Date(dd/mm/yyyy)	UP (cm)	dUP (cm)
31/05/2000	4.9	0.5
07/06/2000	4.9	0.3
14/06/2000	5.4	0.1
21/06/2000	5.4	0.3
28/06/2000	5.7	0.3
05/07/2000	6.4	0.1
12/07/2000	6.6	0.4
19/07/2000	6.5	0.2
26/07/2000	7.2	0.2
02/08/2000	7.6	0.3
09/08/2000	7.7	0.2
16/08/2000	7.6	0.4
23/08/2000	8.0	0.1
30/08/2000	7.8	0.5
06/09/2000	7.7	0.5
13/09/2000	8.0	0.2
20/09/2000	7.7	0.2
27/09/2000	8.0	0.3
04/10/2000	8.0	0.3
11/10/2000	7.7	0.3
18/10/2000	7.9	0.4
25/10/2000	7.6	0.2
01/11/2000	7.8	0.3
08/11/2000	7.4	0.2
15/11/2000	6.9	0.2
22/11/2000	6.8	0.3
29/11/2000	6.9	0.4
06/12/2000	6.9	0.1
13/12/2000	6.7	0.2
20/12/2000	6.6	0.1
27/12/2000	6.0	0.2
03/01/2001	6.0	0.3
10/01/2001	5.9	0.2
17/01/2001	6.3	0.2
24/01/2001	6.0	0.1
31/01/2001	6.0	0.2
07/02/2001	5.7	0.1
14/02/2001	5.6	0.2
21/02/2001	5.7	0.2
28/02/2001	5.4	0.2
14/03/2001	5.0	0.2
21/03/2001	5.0	0.2
28/03/2001	4.8	0.4
18/04/2001	4.6	0.2
02/05/2001	4.2	0.1
09/05/2001	4.4	0.1
16/05/2001	4.2	0.2
23/05/2001	4.1	0.2
30/05/2001	4.3	0.2
06/06/2001	3.9	0.1
13/06/2001	4.0	0.2
20/06/2001	3.8	0.2
27/06/2001	3.6	0.2
04/07/2001	3.8	0.1
11/07/2001	3.8	0.2
18/07/2001	3.5	0.2
25/07/2001	3.8	0.1
01/08/2001	4.0	0.2
08/08/2001	3.8	0.3
15/08/2001	3.6	0.2
22/08/2001	3.8	0.3
29/08/2001	3.7	0.2

05/09/2001	3.6	0.3
12/09/2001	3.1	0.2
19/09/2001	3.3	0.1
26/09/2001	2.8	0.3
03/10/2001	3.0	0.2
10/10/2001	2.9	0.2
17/10/2001	2.7	0.2
24/10/2001	2.8	0.3
07/11/2001	2.5	0.3
14/11/2001	2.5	0.2
21/11/2001	2.4	0.1
28/11/2001	2.3	0.2
05/12/2001	2.3	0.1
12/12/2001	2.2	0.2
19/12/2001	2.2	0.1
26/12/2001	2.2	0.1
13/02/2002	1.6	0.1
20/02/2002	1.5	0.3
27/02/2002	1.6	0.1
27/03/2002	1.0	0.2
24/04/2002	0.8	0.2
01/05/2002	0.6	0.1
08/05/2002	0.6	0.1
15/05/2002	0.6	0.2
22/05/2002	0.7	0.2
29/05/2002	0.6	0.2
05/06/2002	0.8	0.3
19/06/2002	1.3	0.2
26/06/2002	1.6	0.2
03/07/2002	1.4	0.2
10/07/2002	1.8	0.2
17/07/2002	1.5	0.4
24/07/2002	1.5	0.1
31/07/2002	1.5	0.3
07/08/2002	1.5	0.5
14/08/2002	1.5	0.2
21/08/2002	1.6	0.4
28/08/2002	1.0	0.1
04/09/2002	0.9	0.2
11/09/2002	0.8	0.2
18/09/2002	0.9	0.2
25/09/2002	0.5	0.3
02/10/2002	0.4	0.1
09/10/2002	0.8	0.2
16/10/2002	0.6	0.2
23/10/2002	0.2	0.3
30/10/2002	0.3	0.2
13/11/2002	0.4	0.3
20/11/2002	0.2	0.3
27/11/2002	0.3	0.3
04/12/2002	0.4	0.2
11/12/2002	0.1	0.2
18/12/2002	0.4	0.1
25/12/2002	0.7	0.3
01/01/2003	1.0	0.2
08/01/2003	1.3	0.2
15/01/2003	1.0	0.1
22/01/2003	0.9	0.2
29/01/2003	1.3	0.1
05/02/2003	1.3	0.2
12/02/2003	0.8	0.2
19/02/2003	0.9	0.1
26/02/2003	0.8	0.1
05/03/2003	0.7	0.1
12/03/2003	0.7	0.1
19/03/2003	0.8	0.1
26/03/2003	0.8	0.2
02/04/2003	0.8	0.2
09/04/2003	0.8	0.2
16/04/2003	0.8	0.2

23/04/2003	0.9	0.1
30/04/2003	1.0	0.1
07/05/2003	1.0	0.2
14/05/2003	1.0	0.2
21/05/2003	1.1	0.2
28/05/2003	1.1	0.2
04/06/2003	1.1	0.1
11/06/2003	1.1	0.2
18/06/2003	1.3	0.3
25/06/2003	1.1	0.1
02/07/2003	1.0	0.2
09/07/2003	1.0	0.2
16/07/2003	1.3	0.2
23/07/2003	1.3	0.3
30/07/2003	1.3	0.2
06/08/2003	1.6	0.4
13/08/2003	1.3	0.2
20/08/2003	1.0	0.2
27/08/2003	0.8	0.1
03/09/2003	1.1	0.1
10/09/2003	0.9	0.2
17/09/2003	0.9	0.2
24/09/2003	0.9	0.2
01/10/2003	1.1	0.3
08/10/2003	1.0	0.1
15/10/2003	0.9	0.2
22/10/2003	0.9	0.3
29/10/2003	0.8	0.2
05/11/2003	0.5	0.2
12/11/2003	0.7	0.1
19/11/2003	0.6	0.2
26/11/2003	0.9	0.3
03/12/2003	0.7	0.1
10/12/2003	0.7	0.1
17/12/2003	0.9	0.2
24/12/2003	0.8	0.1
31/12/2003	0.8	0.1
14/01/2004	0.5	0.2
21/01/2004	0.9	0.2
28/01/2004	0.6	0.1
04/02/2004	0.4	0.1
11/02/2004	0.4	0.1
18/02/2004	0.6	0.1
25/02/2004	0.6	0.2
03/03/2004	0.5	0.2
10/03/2004	0.3	0.2
17/03/2004	0.3	0.1
24/03/2004	0.5	0.2
31/03/2004	0.4	0.1
14/04/2004	0.4	0.1
21/04/2004	0.3	0.3
28/04/2004	0.6	0.3
05/05/2004	0.4	0.2
12/05/2004	0.6	0.1
26/05/2004	0.4	0.2
02/06/2004	0.4	0.1
16/06/2004	0.4	0.3
23/06/2004	0.5	0.2
30/06/2004	0.5	0.2
07/07/2004	0.4	0.1
14/07/2004	0.4	0.2
21/07/2004	0.5	0.3
28/07/2004	0.5	0.3
11/08/2004	0.4	0.3
18/08/2004	0.3	0.2
25/08/2004	0.3	0.2
01/09/2004	0.2	0.2
08/09/2004	0.4	0.2
15/09/2004	0.1	0.2
22/09/2004	0.2	0.2

29/09/2004	0.0	0.2
06/10/2004	0.2	0.1
13/10/2004	0.2	0.5
20/10/2004	0.1	0.2
27/10/2004	0.0	0.1
03/11/2004	0.5	0.2
10/11/2004	0.3	0.2
17/11/2004	0.2	0.1
24/11/2004	0.1	0.1
01/12/2004	0.4	0.3
08/12/2004	0.3	0.1
15/12/2004	0.3	0.1
22/12/2004	0.3	0.1
29/12/2004	0.5	0.3
05/01/2005	0.2	0.1
12/01/2005	0.4	0.3
19/01/2005	0.2	0.2
26/01/2005	0.6	0.1
02/02/2005	0.2	0.1
09/02/2005	0.1	0.2
16/02/2005	0.8	0.1
23/02/2005	0.4	0.1
02/03/2005	0.2	0.1
09/03/2005	0.2	0.1
16/03/2005	0.1	0.1
23/03/2005	0.1	0.1
30/03/2005	0.1	0.2
06/04/2005	0.3	0.1
13/04/2005	0.2	0.2
20/04/2005	0.1	0.2
27/04/2005	0.2	0.2
04/05/2005	0.3	0.3
11/05/2005	0.4	0.2
18/05/2005	0.5	0.2
25/05/2005	0.5	0.2
08/06/2005	0.7	0.2
15/06/2005	0.3	0.1
22/06/2005	0.3	0.2
29/06/2005	0.6	0.3
06/07/2005	0.6	0.2
13/07/2005	0.6	0.3
20/07/2005	0.5	0.1
27/07/2005	0.6	0.1
03/08/2005	0.6	0.2
10/08/2005	0.6	0.2
17/08/2005	0.7	0.3
24/08/2005	0.7	0.2
31/08/2005	0.7	0.2
07/09/2005	0.7	0.2
14/09/2005	0.7	0.2
21/09/2005	0.7	0.2
28/09/2005	0.7	0.1
05/10/2005	0.5	0.2
12/10/2005	0.5	0.2
19/10/2005	0.5	0.1
26/10/2005	0.6	0.1
02/11/2005	0.7	0.1
09/11/2005	0.7	0.2
16/11/2005	1.0	0.2
23/11/2005	0.9	0.2
30/11/2005	1.1	0.1
21/12/2005	1.1	0.1
28/12/2005	1.4	0.2
11/01/2006	1.1	0.1
18/01/2006	1.5	0.2
25/01/2006	1.5	0.2
01/02/2006	1.5	0.2
22/02/2006	1.7	0.1
01/03/2006	1.7	0.1
08/03/2006	1.9	0.3

15/03/2006	2.0	0.1
22/03/2006	2.0	0.1
29/03/2006	2.0	0.1
05/04/2006	2.2	0.2
19/04/2006	2.2	0.1
26/04/2006	2.7	0.2
03/05/2006	2.4	0.2
10/05/2006	2.3	0.2
17/05/2006	2.6	0.1
24/05/2006	2.7	0.2
31/05/2006	3.0	0.3
07/06/2006	2.6	0.2
14/06/2006	2.7	0.1
21/06/2006	2.7	0.3
28/06/2006	2.9	0.1
05/07/2006	2.8	0.3
12/07/2006	3.0	0.2
02/08/2006	3.2	0.2
09/08/2006	3.2	0.3
16/08/2006	3.4	0.2
23/08/2006	3.7	0.2
30/08/2006	3.7	0.3
06/09/2006	4.2	0.1
13/09/2006	4.3	0.2
20/09/2006	4.1	0.3
27/09/2006	4.1	0.2
04/10/2006	4.3	0.3
11/10/2006	4.1	0.2
18/10/2006	4.5	0.4
25/10/2006	4.7	0.2
01/11/2006	4.9	0.2
08/11/2006	4.7	0.2
15/11/2006	4.6	0.1
22/11/2006	4.5	0.1
29/11/2006	4.6	0.1
06/12/2006	4.6	0.3
13/12/2006	4.7	0.1
20/12/2006	5.0	0.2
27/12/2006	4.8	0.1
03/01/2007	4.9	0.1
10/01/2007	4.7	0.1
17/01/2007	4.7	0.2
24/01/2007	5.0	0.1
31/01/2007	4.8	0.1
07/02/2007	4.6	0.3
14/02/2007	4.5	0.1
21/02/2007	4.5	0.2
28/02/2007	4.3	0.2
07/03/2007	4.5	0.1
14/03/2007	4.4	0.1
21/03/2007	4.5	0.2
28/03/2007	4.1	0.2
04/04/2007	4.3	0.2
11/04/2007	4.0	0.1
02/05/2007	4.0	0.1
09/05/2007	3.8	0.1
16/05/2007	3.9	0.2
23/05/2007	3.9	0.2
30/05/2007	3.4	0.3
06/06/2007	3.5	0.3
13/06/2007	3.5	0.2
20/06/2007	3.5	0.2
27/06/2007	3.6	0.3
04/07/2007	3.5	0.2
11/07/2007	3.6	0.2
18/07/2007	3.4	0.2
25/07/2007	3.6	0.3
08/08/2007	3.2	0.3
15/08/2007	2.9	0.2
22/08/2007	3.3	0.3

29/08/2007	3.4	0.2
05/09/2007	3.0	0.2
19/09/2007	2.9	0.2
26/09/2007	2.9	0.2
17/10/2007	2.6	0.2
24/10/2007	2.8	0.2
31/10/2007	2.9	0.2
07/11/2007	2.8	0.2
14/11/2007	3.1	0.2
21/11/2007	2.8	0.2
28/11/2007	3.1	0.1
05/12/2007	2.9	0.2
12/12/2007	3.5	0.1
19/12/2007	3.1	0.1
26/12/2007	3.0	0.1
02/01/2008	3.4	0.2
09/01/2008	3.6	0.1
16/01/2008	3.4	0.1
30/01/2008	3.7	0.3
06/02/2008	3.4	0.1
20/02/2008	3.3	0.1
27/02/2008	3.2	0.1
05/03/2008	3.5	0.2
12/03/2008	3.3	0.1
26/03/2008	3.2	0.2
02/04/2008	3.2	0.4
09/04/2008	3.1	0.2
16/04/2008	3.2	0.3
23/04/2008	3.2	0.3
30/04/2008	3.3	0.1
07/05/2008	3.2	0.2
14/05/2008	3.4	0.1
21/05/2008	3.7	0.2
28/05/2008	3.5	0.3
04/06/2008	3.5	0.3
11/06/2008	3.5	0.2
18/06/2008	3.4	0.3
25/06/2008	3.4	0.3
02/07/2008	3.4	0.1
09/07/2008	3.2	0.1
16/07/2008	3.3	0.1
23/07/2008	3.6	0.2
30/07/2008	3.7	0.2
06/08/2008	3.6	0.2
13/08/2008	3.8	0.2
20/08/2008	3.5	0.3
27/08/2008	3.9	0.2
03/09/2008	3.6	0.2
10/09/2008	3.5	0.3
17/09/2008	3.8	0.2
24/09/2008	3.6	0.1
01/10/2008	3.8	0.1
08/10/2008	3.5	0.2
15/10/2008	3.7	0.1
22/10/2008	3.7	0.1
29/10/2008	3.8	0.2
05/11/2008	3.9	0.1
12/11/2008	3.9	0.2
19/11/2008	4.0	0.3
26/11/2008	4.2	0.1
03/12/2008	4.2	0.2
10/12/2008	4.2	0.1
17/12/2008	4.2	0.1
24/12/2008	4.1	0.1
31/12/2008	4.3	0.1
07/01/2009	4.5	0.1
14/01/2009	4.3	0.2
21/01/2009	4.7	0.1
28/01/2009	4.0	0.1
04/02/2009	4.0	0.1

11/02/2009	4.2	0.1
18/02/2009	4.3	0.1
25/02/2009	4.4	0.1
04/03/2009	4.6	0.1
11/03/2009	4.5	0.2
18/03/2009	4.5	0.1
25/03/2009	4.7	0.2
01/04/2009	4.8	0.2
08/04/2009	4.6	0.1
15/04/2009	4.7	0.1
22/04/2009	4.8	0.2
29/04/2009	4.6	0.2
06/05/2009	4.8	0.1
13/05/2009	5.0	0.1
20/05/2009	5.2	0.2
27/05/2009	5.2	0.1
03/06/2009	5.3	0.2
10/06/2009	5.1	0.3
17/06/2009	5.6	0.3
24/06/2009	5.6	0.4
01/07/2009	5.6	0.2
08/07/2009	5.2	0.2
22/07/2009	5.5	0.2
29/07/2009	5.4	0.2
05/08/2009	5.5	0.2
12/08/2009	5.2	0.2
19/08/2009	5.3	0.1
26/08/2009	5.3	0.2
02/09/2009	5.5	0.3
09/09/2009	5.9	0.2
16/09/2009	5.6	0.2
23/09/2009	5.6	0.2
30/09/2009	5.5	0.1
07/10/2009	5.4	0.1
14/10/2009	5.6	0.3
21/10/2009	5.5	0.1
28/10/2009	5.1	0.1
04/11/2009	5.3	0.3
11/11/2009	5.4	0.2
18/11/2009	5.2	0.1
25/11/2009	5.4	0.3
02/12/2009	5.1	0.2
09/12/2009	5.0	0.1
16/12/2009	5.4	0.1
23/12/2009	5.0	0.4
30/12/2009	5.1	0.2
06/01/2010	5.4	0.2
13/01/2010	4.8	0.2
20/01/2010	5.0	0.2
27/01/2010	5.2	0.1
03/02/2010	5.2	0.0
10/02/2010	5.4	0.1
24/02/2010	4.9	0.2
03/03/2010	5.2	0.2
10/03/2010	5.3	0.2
17/03/2010	5.0	0.1
24/03/2010	5.0	0.2
31/03/2010	5.0	0.3
07/04/2010	5.0	0.1
14/04/2010	4.9	0.2
21/04/2010	5.0	0.2
28/04/2010	5.2	0.1
05/05/2010	5.1	0.1
12/05/2010	5.0	0.1
19/05/2010	5.2	0.3
02/06/2010	5.2	0.1
09/06/2010	5.2	0.2
16/06/2010	5.5	0.1
23/06/2010	5.4	0.2
30/06/2010	5.7	0.3

07/07/2010	5.7	0.2
14/07/2010	5.8	0.2
21/07/2010	6.3	0.5
28/07/2010	6.0	0.4
04/08/2010	6.3	0.2
11/08/2010	6.4	0.2
18/08/2010	6.4	0.1
25/08/2010	6.3	0.1
01/09/2010	6.5	0.2
08/09/2010	6.5	0.3
15/09/2010	6.5	0.1
22/09/2010	6.3	0.2
29/09/2010	6.5	0.3
06/10/2010	6.5	0.3
13/10/2010	6.9	0.1
20/10/2010	6.7	0.2
03/11/2010	6.8	0.1
10/11/2010	6.8	0.2
17/11/2010	6.5	0.1
24/11/2010	7.1	0.2
01/12/2010	7.2	0.2
08/12/2010	6.8	0.1
15/12/2010	6.9	0.2
22/12/2010	6.8	0.1
29/12/2010	6.9	0.2
05/01/2011	6.7	0.1
12/01/2011	6.9	0.1
19/01/2011	6.9	0.1
26/01/2011	6.8	0.1
02/02/2011	6.9	0.2
09/02/2011	6.8	0.1
16/02/2011	7.2	0.1
23/02/2011	6.9	0.1
02/03/2011	6.8	0.1
09/03/2011	6.5	0.1
16/03/2011	6.9	0.1
23/03/2011	6.7	0.1
30/03/2011	6.6	0.2
06/04/2011	6.8	0.2
13/04/2011	6.8	0.2
20/04/2011	7.0	0.1
27/04/2011	7.6	0.2
04/05/2011	7.6	0.2
11/05/2011	7.8	0.3
18/05/2011	8.2	0.1
25/05/2011	8.5	0.2
01/06/2011	8.5	0.2
08/06/2011	9.0	0.2
15/06/2011	8.8	0.2
22/06/2011	9.2	0.1
29/06/2011	9.3	0.2
06/07/2011	8.9	0.1
20/07/2011	9.0	0.3
27/07/2011	9.2	0.3
03/08/2011	9.2	0.1
10/08/2011	9.4	0.2
17/08/2011	9.4	0.1
24/08/2011	9.4	0.2
31/08/2011	9.7	0.3
07/09/2011	9.8	0.2
14/09/2011	9.7	0.1
21/09/2011	10.1	0.1
05/10/2011	10.3	0.2
12/10/2011	10.5	0.3
19/10/2011	10.8	0.5
26/10/2011	10.3	0.3
02/11/2011	10.4	0.1
09/11/2011	10.5	0.1
16/11/2011	10.8	1.7
23/11/2011	10.7	0.1

30/11/2011	10.9	0.2
07/12/2011	10.9	0.2
14/12/2011	10.7	0.3
21/12/2011	10.9	0.1
28/12/2011	10.6	0.2
04/01/2012	10.8	0.1
11/01/2012	10.7	0.2
18/01/2012	11.0	0.2
25/01/2012	11.2	0.1
01/02/2012	11.2	0.1
08/02/2012	11.1	0.1
15/02/2012	11.1	0.2
22/02/2012	11.4	0.1
29/02/2012	11.4	0.2
07/03/2012	11.7	0.2
14/03/2012	11.4	0.3
21/03/2012	11.6	0.1
28/03/2012	11.7	0.2
04/04/2012	11.5	0.2
18/04/2012	11.6	0.2
25/04/2012	11.5	0.2
02/05/2012	11.7	0.4
09/05/2012	11.8	0.3
16/05/2012	12.1	0.1
23/05/2012	12.1	0.2
30/05/2012	12.3	0.2
06/06/2012	12.4	0.1
13/06/2012	12.3	0.3
20/06/2012	12.6	0.2
27/06/2012	13.0	0.2
04/07/2012	13.0	0.2
11/07/2012	13.4	0.2
18/07/2012	13.6	0.2
25/07/2012	13.9	0.2
01/08/2012	14.5	0.3
08/08/2012	15.2	0.3
15/08/2012	15.5	0.3
22/08/2012	15.9	0.3
29/08/2012	16.4	0.3
05/09/2012	16.9	0.2
12/09/2012	17.0	0.2
19/09/2012	17.2	0.2
26/09/2012	17.3	0.3
03/10/2012	17.5	0.2
10/10/2012	17.7	0.2
17/10/2012	17.6	0.2
24/10/2012	18.0	0.3
31/10/2012	17.8	0.2
07/11/2012	17.8	0.4
14/11/2012	18.4	0.2
21/11/2012	18.1	0.2
28/11/2012	17.9	0.1
05/12/2012	18.0	0.3
12/12/2012	18.5	0.2
19/12/2012	19.6	0.2
26/12/2012	19.6	0.3
02/01/2013	20.3	0.1
09/01/2013	20.8	0.1
16/01/2013	21.2	0.2
23/01/2013	21.3	0.3
30/01/2013	21.6	0.3
06/02/2013	21.9	0.2
13/02/2013	22.2	0.1
20/02/2013	22.2	0.1
27/02/2013	22.2	0.1
06/03/2013	21.9	0.1
13/03/2013	22.2	0.1
20/03/2013	22.1	0.2
27/03/2013	22.7	0.1
03/04/2013	22.4	0.2

10/04/2013	22.6	0.2
17/04/2013	23.0	0.1
24/04/2013	23.2	0.2
01/05/2013	23.0	0.1
08/05/2013	23.0	0.2
15/05/2013	23.2	0.2
22/05/2013	22.7	0.3
29/05/2013	22.7	0.3
05/06/2013	23.0	0.1
12/06/2013	23.0	0.3
19/06/2013	22.9	0.2
26/06/2013	23.1	0.2
03/07/2013	22.9	0.2
10/07/2013	22.9	0.1
17/07/2013	22.6	0.2
24/07/2013	23.0	0.2
31/07/2013	22.9	0.3
07/08/2013	23.1	0.2
14/08/2013	22.6	0.2
21/08/2013	22.5	0.1
28/08/2013	22.6	0.1
04/09/2013	22.7	0.2
11/09/2013	22.9	0.3
18/09/2013	22.8	0.3
25/09/2013	22.3	0.2
02/10/2013	22.6	0.2
09/10/2013	22.6	0.4
16/10/2013	22.4	0.1
23/10/2013	22.4	0.1
30/10/2013	22.5	0.2
06/11/2013	22.7	0.3
13/11/2013	22.5	0.1
20/11/2013	22.5	0.2
27/11/2013	22.6	0.1
04/12/2013	22.6	0.2
11/12/2013	22.5	0.1
18/12/2013	22.3	0.1
25/12/2013	22.4	0.1
01/01/2014	22.2	0.1
08/01/2014	22.3	0.1
15/01/2014	22.6	0.2
22/01/2014	22.7	0.2
29/01/2014	22.8	0.2
05/02/2014	22.7	0.1
12/02/2014	22.7	0.2
19/02/2014	22.8	0.1
26/02/2014	22.3	0.1
05/03/2014	22.6	0.3
12/03/2014	22.8	0.1
19/03/2014	22.7	0.1
26/03/2014	23.1	0.1
02/04/2014	22.7	0.2
09/04/2014	22.8	0.1
16/04/2014	23.1	0.1
23/04/2014	23.1	0.1
30/04/2014	22.8	0.1
07/05/2014	22.9	0.1
14/05/2014	23.2	0.2
21/05/2014	23.1	0.1
28/05/2014	23.4	0.2
04/06/2014	23.4	0.2
11/06/2014	23.4	0.2
18/06/2014	23.5	0.3
25/06/2014	23.4	0.3
02/07/2014	23.5	0.1
09/07/2014	23.8	0.4
16/07/2014	23.7	0.2
23/07/2014	24.3	0.4
30/07/2014	24.4	0.3
06/08/2014	24.6	0.2

13/08/2014	24.6	0.3
20/08/2014	24.9	0.2
27/08/2014	24.9	0.4
03/09/2014	25.5	0.3
10/09/2014	25.6	0.2
17/09/2014	25.8	0.2
24/09/2014	26.5	0.2
01/10/2014	26.6	0.1
08/10/2014	26.5	0.1
15/10/2014	26.9	0.1
22/10/2014	27.1	0.2
29/10/2014	27.1	0.2
05/11/2014	27.1	0.3
12/11/2014	27.0	0.2
19/11/2014	27.3	0.3
26/11/2014	27.4	0.1
03/12/2014	27.4	0.1
10/12/2014	27.4	0.1
17/12/2014	27.5	0.1
24/12/2014	27.6	0.1
31/12/2014	28.4	0.2
07/01/2015	28.0	0.2
14/01/2015	28.0	0.2
21/01/2015	28.7	0.2
28/01/2015	28.7	0.2
04/02/2015	28.6	0.1
11/02/2015	28.7	0.2
18/02/2015	28.8	0.1
25/02/2015	28.7	0.1
04/03/2015	28.6	0.4
11/03/2015	28.7	0.1
18/03/2015	29.0	0.1
25/03/2015	29.0	0.1
01/04/2015	29.2	0.1
08/04/2015	29.0	0.1
15/04/2015	29.2	0.1
22/04/2015	29.4	0.1
29/04/2015	29.4	0.2
06/05/2015	29.6	0.1
13/05/2015	30.0	0.2
20/05/2015	29.9	0.1
27/05/2015	29.8	0.1
03/06/2015	29.9	0.2
10/06/2015	30.0	0.1
17/06/2015	30.3	0.2
24/06/2015	30.4	0.1
01/07/2015	30.5	0.2
08/07/2015	30.8	0.2
15/07/2015	30.8	0.1
22/07/2015	30.5	0.3
29/07/2015	30.5	0.2
05/08/2015	30.7	0.4
12/08/2015	30.8	0.1
19/08/2015	31.4	0.1
26/08/2015	31.4	0.1
02/09/2015	31.8	0.2
09/09/2015	32.3	0.2
16/09/2015	32.8	0.2
23/09/2015	32.9	0.2
30/09/2015	33.4	0.2
07/10/2015	33.2	0.3
14/10/2015	33.1	0.2
21/10/2015	33.3	0.3
28/10/2015	33.0	0.2
04/11/2015	33.1	0.1
11/11/2015	33.3	0.1
18/11/2015	33.5	0.2
25/11/2015	33.6	0.2
02/12/2015	33.6	0.1
09/12/2015	33.7	0.2

16/12/2015	33.7	0.1
23/12/2015	34.0	0.0
30/12/2015	34.1	0.1
06/01/2016	34.1	0.3
13/01/2016	34.4	0.3
20/01/2016	34.7	0.1
27/01/2016	34.6	0.1
03/02/2016	34.8	0.2
10/02/2016	34.5	0.2
17/02/2016	35.0	0.1
24/02/2016	35.3	0.2
02/03/2016	35.9	0.3
09/03/2016	35.2	0.2
16/03/2016	35.7	0.1
23/03/2016	35.6	0.1
30/03/2016	35.5	0.1
06/04/2016	36.0	0.2
13/04/2016	36.3	0.2
20/04/2016	36.6	0.2
27/04/2016	37.2	0.2
04/05/2016	37.5	0.1
11/05/2016	37.9	0.1
18/05/2016	38.2	0.2
25/05/2016	38.4	0.1
01/06/2016	38.5	0.2
08/06/2016	38.6	0.1
15/06/2016	38.8	0.3
22/06/2016	39.0	0.3
29/06/2016	39.0	0.3
06/07/2016	39.7	0.2
